# Supplementary material for: Dominant nitrogen metabolisms of a warm, seasonally anoxic freshwater ecosystem revealed using genome resolved metatranscriptomics
Source: mSystems. 2024 Jan 23;9(2):e01059-23. doi: 10.1128/msystems.01059-23 (PMC10878078; doi:10.1128/msystems.01059-23)
Supplement: Supplemental Materials — Tables S1 and S2 and Fig. S1-S5. [file msystems.01059-23-s0003.pdf]

Supplementary Tables

Table S1. Mean ± standard error of geochemical parameters across the three sampling locations.

	NH_4^+ (µM)	NO_3^- (µM)	DOC (µM)	TP (µM)
January (1 m)	38.01 ± 3.19	15.45 ± 0.25	200.75 ± 7.21	0.56 ± 0.03
January (16 m)	39.86 ± 3.38	15.50 ± 0.27	208.75 ± 7.73	0.60 ± 0.21
June (1 m)	3.85 ± 0.67	2.04 ± 0.43	225.26 ± 25.91	1.23 ± 0.40
June (16 m)	27.25 ± 5.18	1.79 ± 0.21	154.87 ± 14.86	0.83 ± 0.28

Table S2. Nitrogen metabolism genes considered in this analysis.

Kegg ID	Gene	Description
K00367	*narB*	assimilatory nitrate reductase
K00372	*nasC, nasA*	assimilatory nitrate reductase
K00366	*nirA*	assimilatory nitrite reductase
K02568	*napB*	Nitrate reductase, cytochrome c-type protein
K10535	*hao*	hydroxylamine oxidase
K00368	*nirK*	nitrite reductase (NO-forming)
K15864	*nirS*	nitrite reductase (NO-forming)
K00374	*narI, narV*	nitrate reductase 1
K04561	*norB*	nitric oxide reductase
K02305	*norC*	nitric oxide reductase
K02588	*nifH*	nitrogenase iron protein
K02586	*nifD*	nitrogenase molybdenum-iron protein
K02591	*nifK*	nitrogenase molybdenum-iron protein
K00376	*nosZ*	nitrous-oxide reductase
K00371	*narH, narY, nxrB*	nitrite oxidoreductase
K00370	*narG, narZ, nxrA*	nitrite oxidoreductase
K02567	*napA*	periplasmic nitrate reductase
K00362	*nirB*	respiratory nitrite reductase
K00363	*nirD*	respiratory nitrite reductase
K03385	*nrfA*	respiratory nitrite reductase
K15876	*nrfH*	respiratory nitrite reductase

Supplementary Figures

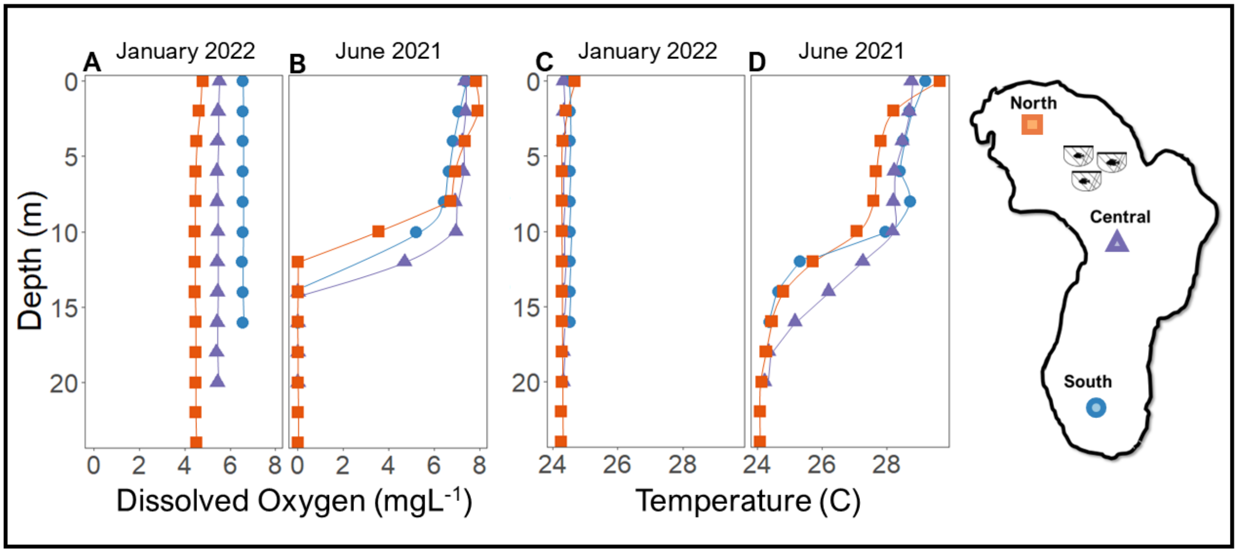

Figure S1. Thermophysical structure in Lake Yojoa. **(A)** Dissolved oxygen at 2 m intervals in January 2022 and June 2021**. (B)** Temperature at 2 m intervals in January 2022 and June 2021.

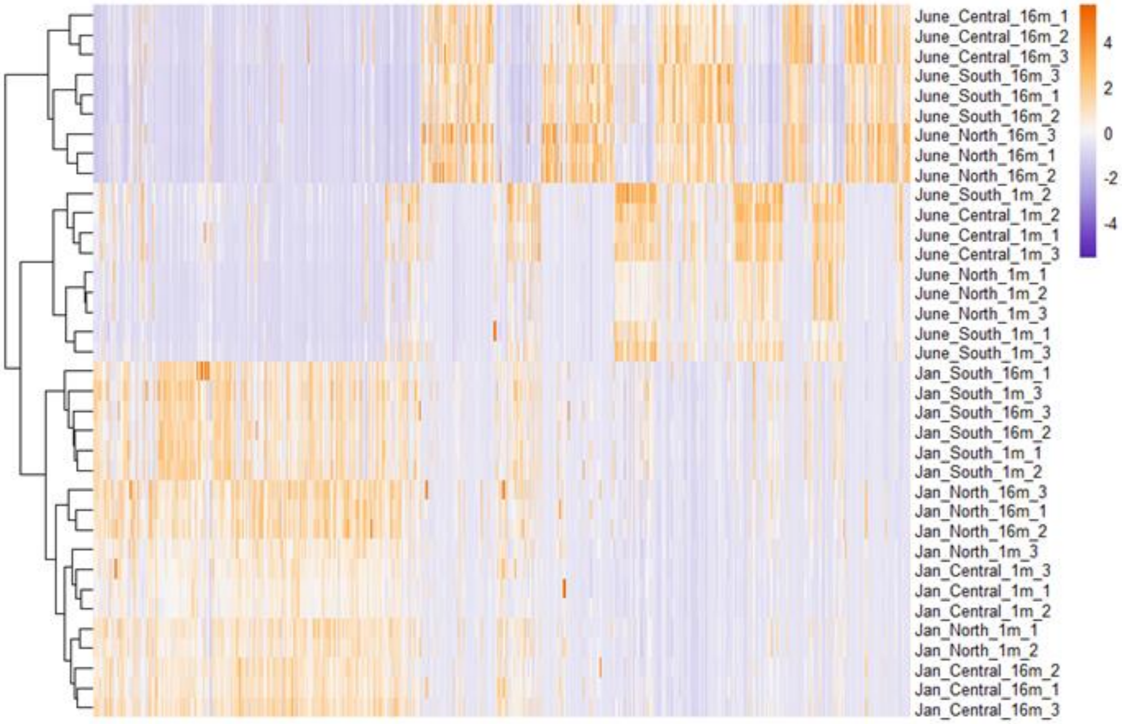

Figure S2. MAG relative expression heatmap with hierarchical clustering.

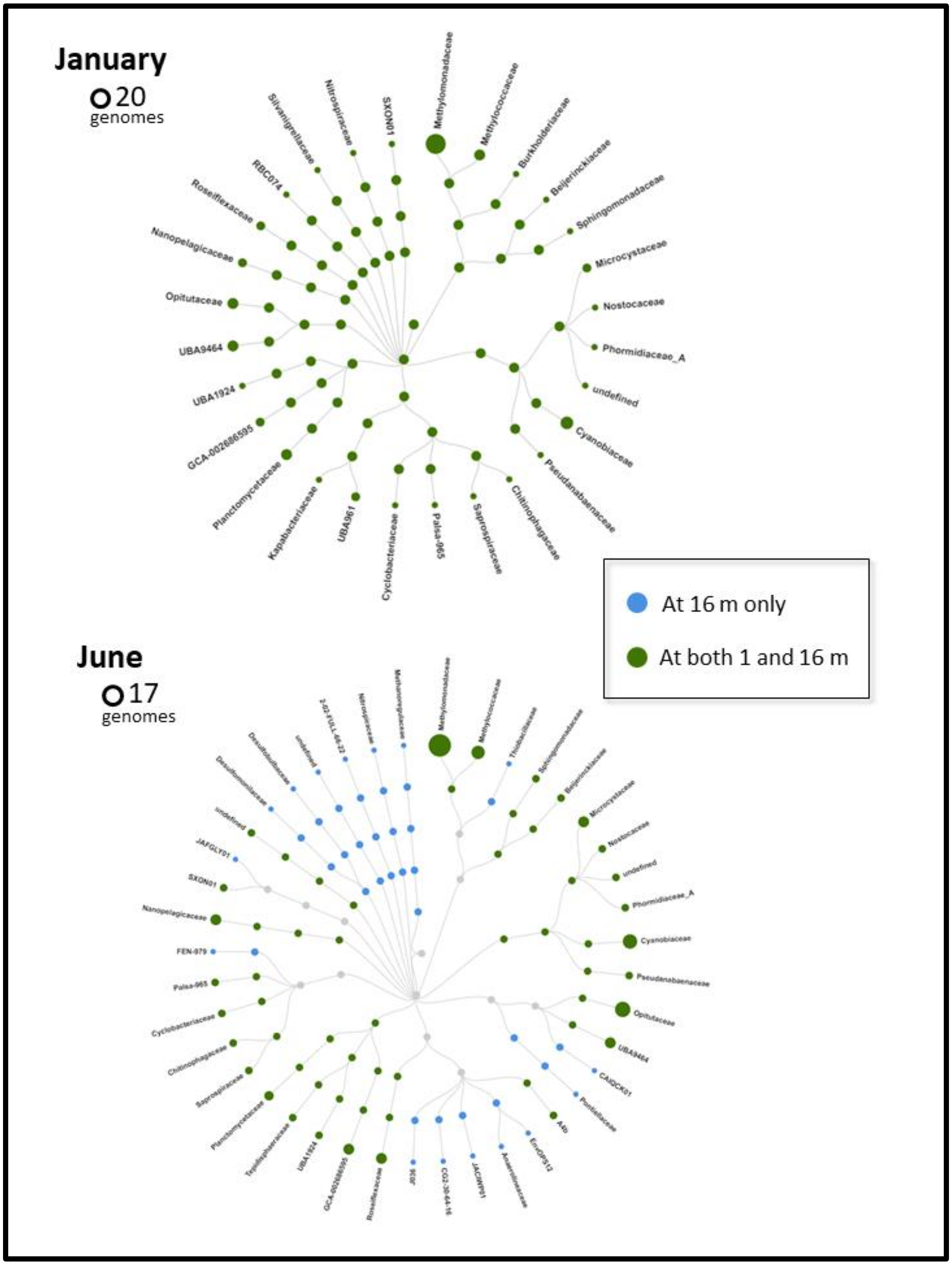

Figure S3. Taxonomic level (Domain, Phylum, Class, Order, Family) assigned by GTDB-tk to genomes expressing N metabolism genes of interest (Table S1) in January (n= 20) and June (n=17).

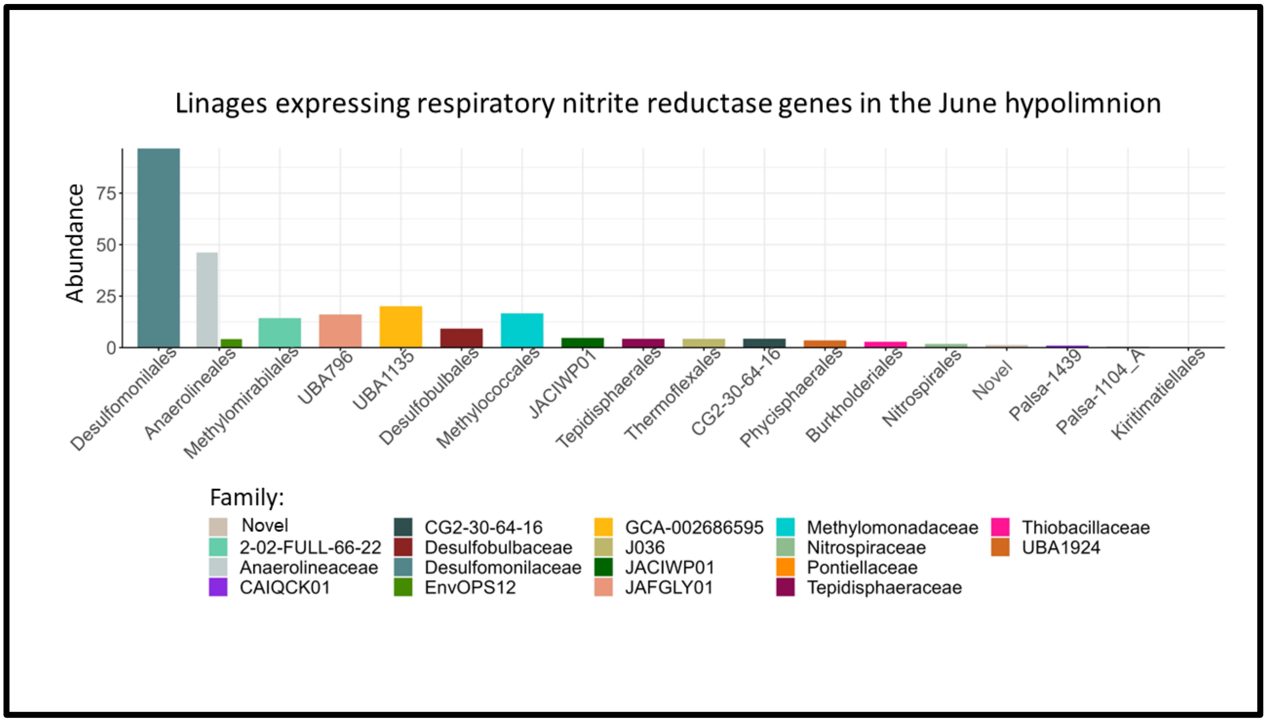

Figure S4. Lineages of MAGs expresing genes related to respiratory nitrite reduction (Table S1). Abundance refers to total expression.

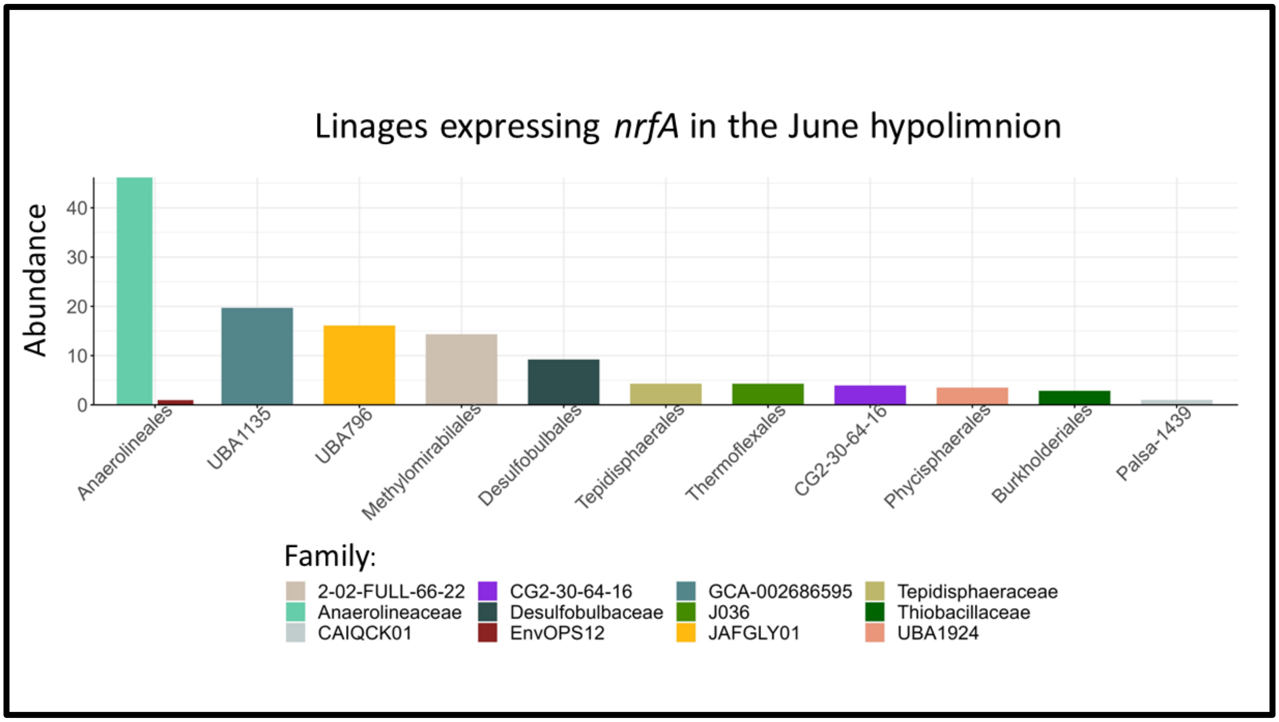

Figure S5. Lineage of MAGs expressing *nrfA* in the June hypolimnion. Abundance refers to total expression.
