# Supplementary material for: Dominant nitrogen metabolisms of a warm, seasonally anoxic freshwater ecosystem revealed using genome resolved metatranscriptomics
Source: mSystems. 2024 Jan 23;9(2):e01059-23. doi: 10.1128/msystems.01059-23 (PMC10878078; doi:10.1128/msystems.01059-23)
Supplement: Table S3 — Supplemental MAG information. [file msystems.01059-23-s0004.pdf]

Table S3 Supplemental MAG information.

Accession	Sample Name	SPUID	Organism	Tax ID	BioProject
SAMN37499007	Yojoa_01_2020_R_epi_A_bin.111	Yojoa_01_2020_R_epi_A_bin.111	Planctomycetota bacterium	2026780	PRJNA946291
SAMN37499008	Yojoa_01_2020_R_epi_A_bin.112	Yojoa_01_2020_R_epi_A_bin.112	Bacteroidota bacterium	1898104	PRJNA946291
SAMN37499009	Yojoa_01_2020_R_epi_A_bin.114	Yojoa_01_2020_R_epi_A_bin.114	Pseudomonadota bacterium	1977087	PRJNA946291
SAMN37499010	Yojoa_01_2020_R_epi_A_bin.115	Yojoa_01_2020_R_epi_A_bin.115	Actinomycetota bacterium	2900548	PRJNA946291
SAMN37499011	Yojoa_01_2020_R_epi_A_bin.12	Yojoa_01_2020_R_epi_A_bin.12	Nitrospirota bacterium	2026887	PRJNA946291
SAMN37499012	Yojoa_01_2020_R_epi_A_bin.38	Yojoa_01_2020_R_epi_A_bin.38	Verrucomicrobiota bacterium	2026799	PRJNA946291
SAMN37499013	Yojoa_01_2020_R_epi_A_bin.39	Yojoa_01_2020_R_epi_A_bin.39	Pseudomonadota bacterium	1977087	PRJNA946291
SAMN37499014	Yojoa_01_2020_R_epi_A_bin.44	Yojoa_01_2020_R_epi_A_bin.44	Planctomycetota bacterium	2026780	PRJNA946291
SAMN37499015	Yojoa_01_2020_R_epi_A_bin.45	Yojoa_01_2020_R_epi_A_bin.45	Verrucomicrobiota bacterium	2026799	PRJNA946291
SAMN37499016	Yojoa_01_2020_R_epi_A_bin.59	Yojoa_01_2020_R_epi_A_bin.59	Planctomycetota bacterium	2026780	PRJNA946291
SAMN37499017	Yojoa_01_2020_R_epi_A_bin.68	Yojoa_01_2020_R_epi_A_bin.68	Bacteroidota bacterium	1898104	PRJNA946291
SAMN37499018	Yojoa_01_2020_R_epi_A_bin.9	Yojoa_01_2020_R_epi_A_bin.9	Gemmatimonadota bacterium	2026742	PRJNA946291
SAMN37499019	Yojoa_06_2019_Q_hypo_A_bin.10	Yojoa_06_2019_Q_hypo_A_bin.10	Chloroflexota bacterium	2026724	PRJNA946291
SAMN37499020	Yojoa_06_2019_Q_hypo_A_bin.104	Yojoa_06_2019_Q_hypo_A_bin.104	Pseudomonadota bacterium	1977087	PRJNA946291
SAMN37499021	Yojoa_06_2019_Q_hypo_A_bin.110	Yojoa_06_2019_Q_hypo_A_bin.110	Planctomycetota bacterium	2026780	PRJNA946291
SAMN37499022	Yojoa_06_2019_Q_hypo_A_bin.111	Yojoa_06_2019_Q_hypo_A_bin.111	Methanotrichaceae archaeon	2595067	PRJNA946291
SAMN37499023	Yojoa_06_2019_Q_hypo_A_bin.114	Yojoa_06_2019_Q_hypo_A_bin.114	Methanomicrobiales archaeon	2184052	PRJNA946291
SAMN37499024	Yojoa_06_2019_Q_hypo_A_bin.119	Yojoa_06_2019_Q_hypo_A_bin.119	Planctomycetota bacterium	2026780	PRJNA946291
SAMN37499025	Yojoa_06_2019_Q_hypo_A_bin.125	Yojoa_06_2019_Q_hypo_A_bin.125	Bacteroidota bacterium	1898104	PRJNA946291
SAMN37499026	Yojoa_06_2019_Q_hypo_A_bin.128	Yojoa_06_2019_Q_hypo_A_bin.128	Candidatus Parcubacteria bacterium	2762014	PRJNA946291
SAMN37499027	Yojoa_06_2019_Q_hypo_A_bin.136	Yojoa_06_2019_Q_hypo_A_bin.136	Candidatus Hydrogenedentes bacterium	2030809	PRJNA946291
SAMN37499028	Yojoa_06_2019_Q_hypo_A_bin.137	Yojoa_06_2019_Q_hypo_A_bin.137	Methanotrichaceae archaeon	2595067	PRJNA946291
SAMN37499029	Yojoa_06_2019_Q_hypo_A_bin.138	Yojoa_06_2019_Q_hypo_A_bin.138	Actinomycetota bacterium	2900548	PRJNA946291
SAMN37499030	Yojoa_06_2019_Q_hypo_A_bin.143	Yojoa_06_2019_Q_hypo_A_bin.143	Chlamydiota bacterium	2081524	PRJNA946291
SAMN37499031	Yojoa_06_2019_Q_hypo_A_bin.144	Yojoa_06_2019_Q_hypo_A_bin.144	Pseudomonadota bacterium	1977087	PRJNA946291
SAMN37499032	Yojoa_06_2019_Q_hypo_A_bin.149	Yojoa_06_2019_Q_hypo_A_bin.149	bacterium	1869227	PRJNA946291
SAMN37499033	Yojoa_06_2019_Q_hypo_A_bin.15	Yojoa_06_2019_Q_hypo_A_bin.15	Verrucomicrobiota bacterium	2026799	PRJNA946291
SAMN37499034	Yojoa_06_2019_Q_hypo_A_bin.161	Yojoa_06_2019_Q_hypo_A_bin.161	Actinomycetota bacterium	2900548	PRJNA946291
SAMN37499035	Yojoa_06_2019_Q_hypo_A_bin.162	Yojoa_06_2019_Q_hypo_A_bin.162	Nanoarchaeota archaeon	2026764	PRJNA946291
SAMN37499036	Yojoa_06_2019_Q_hypo_A_bin.166	Yojoa_06_2019_Q_hypo_A_bin.166	Verrucomicrobiota bacterium	2026799	PRJNA946291
SAMN37499037	Yojoa_06_2019_Q_hypo_A_bin.167	Yojoa_06_2019_Q_hypo_A_bin.167	Planctomycetota bacterium	2026780	PRJNA946291
SAMN37499038	Yojoa_06_2019_Q_hypo_A_bin.17	Yojoa_06_2019_Q_hypo_A_bin.17	Candidatus Omnitrophota bacterium	2035772	PRJNA946291
SAMN37499039	Yojoa_06_2019_Q_hypo_A_bin.174	Yojoa_06_2019_Q_hypo_A_bin.174	Pseudomonadota bacterium	1977087	PRJNA946291

SAMN37499040	Yojoa_06_2019_Q_hypo_A_bin.176	Yojoa_06_2019_Q_hypo_A_bin.176	Verrucomicrobiota bacterium	2026799	PRJNA946291
SAMN37499041	Yojoa_06_2019_Q_hypo_A_bin.188	Yojoa_06_2019_Q_hypo_A_bin.188	Candidatus Omnitrophota bacterium	2035772	PRJNA946291
SAMN37499042	Yojoa_06_2019_Q_hypo_A_bin.19	Yojoa_06_2019_Q_hypo_A_bin.19	Verrucomicrobiota bacterium	2026799	PRJNA946291
SAMN37499043	Yojoa_06_2019_Q_hypo_A_bin.194	Yojoa_06_2019_Q_hypo_A_bin.194	Candidatus Omnitrophota bacterium	2035772	PRJNA946291
SAMN37499044	Yojoa_06_2019_Q_hypo_A_bin.196	Yojoa_06_2019_Q_hypo_A_bin.196	Bacteroidota bacterium	1898104	PRJNA946291
SAMN37499045	Yojoa_06_2019_Q_hypo_A_bin.197	Yojoa_06_2019_Q_hypo_A_bin.197	Candidatus Diapherotrites archaeon	2026736	PRJNA946291
SAMN37499046	Yojoa_06_2019_Q_hypo_A_bin.201	Yojoa_06_2019_Q_hypo_A_bin.201	Bacteroidota bacterium	1898104	PRJNA946291
SAMN37499047	Yojoa_06_2019_Q_hypo_A_bin.208	Yojoa_06_2019_Q_hypo_A_bin.208	Bacteroidota bacterium	1898104	PRJNA946291
SAMN37499048	Yojoa_06_2019_Q_hypo_A_bin.211	Yojoa_06_2019_Q_hypo_A_bin.211	Verrucomicrobiota bacterium	2026799	PRJNA946291
SAMN37499049	Yojoa_06_2019_Q_hypo_A_bin.213	Yojoa_06_2019_Q_hypo_A_bin.213	Bacteroidota bacterium	1898104	PRJNA946291
SAMN37499050	Yojoa_06_2019_Q_hypo_A_bin.215	Yojoa_06_2019_Q_hypo_A_bin.215	Bacillota bacterium	1879010	PRJNA946291
SAMN37499051	Yojoa_06_2019_Q_hypo_A_bin.225	Yojoa_06_2019_Q_hypo_A_bin.225	Nanoarchaeota archaeon	2026764	PRJNA946291
SAMN37499052	Yojoa_06_2019_Q_hypo_A_bin.227	Yojoa_06_2019_Q_hypo_A_bin.227	Planctomycetota bacterium	2026780	PRJNA946291
SAMN37499053	Yojoa_06_2019_Q_hypo_A_bin.233	Yojoa_06_2019_Q_hypo_A_bin.233	Verrucomicrobiota bacterium	2026799	PRJNA946291
SAMN37499054	Yojoa_06_2019_Q_hypo_A_bin.235	Yojoa_06_2019_Q_hypo_A_bin.235	Actinomycetota bacterium	2900548	PRJNA946291
SAMN37499055	Yojoa_06_2019_Q_hypo_A_bin.27	Yojoa_06_2019_Q_hypo_A_bin.27	Bacteroidota bacterium	1898104	PRJNA946291
SAMN37499056	Yojoa_06_2019_Q_hypo_A_bin.47	Yojoa_06_2019_Q_hypo_A_bin.47	Verrucomicrobiota bacterium	2026799	PRJNA946291
SAMN37499057	Yojoa_06_2019_Q_hypo_A_bin.58	Yojoa_06_2019_Q_hypo_A_bin.58	Verrucomicrobiota bacterium	2026799	PRJNA946291
SAMN37499058	Yojoa_06_2019_Q_hypo_A_bin.59	Yojoa_06_2019_Q_hypo_A_bin.59	Methanoregulaceae archaeon	2485498	PRJNA946291
SAMN37499059	Yojoa_06_2019_Q_hypo_A_bin.62	Yojoa_06_2019_Q_hypo_A_bin.62	Planctomycetota bacterium	2026780	PRJNA946291
SAMN37499060	Yojoa_06_2019_Q_hypo_A_bin.63	Yojoa_06_2019_Q_hypo_A_bin.63	Bacillota bacterium	1879010	PRJNA946291
SAMN37499061	Yojoa_06_2019_Q_hypo_A_bin.64	Yojoa_06_2019_Q_hypo_A_bin.64	Candidatus Omnitrophota bacterium	2035772	PRJNA946291
SAMN37499062	Yojoa_06_2019_Q_hypo_A_bin.71	Yojoa_06_2019_Q_hypo_A_bin.71	Pseudomonadota bacterium	1977087	PRJNA946291
SAMN37499063	Yojoa_06_2019_Q_hypo_A_bin.72	Yojoa_06_2019_Q_hypo_A_bin.72	Candidatus Omnitrophota bacterium	2035772	PRJNA946291
SAMN37499064	Yojoa_06_2019_Q_hypo_A_bin.78	Yojoa_06_2019_Q_hypo_A_bin.78	Methanotrichaceae archaeon	2595067	PRJNA946291
SAMN37499065	Yojoa_06_2019_Q_hypo_A_bin.79	Yojoa_06_2019_Q_hypo_A_bin.79	Chloroflexota bacterium	2026724	PRJNA946291
SAMN37499066	Yojoa_06_2019_Q_hypo_A_bin.91	Yojoa_06_2019_Q_hypo_A_bin.91	Verrucomicrobiota bacterium	2026799	PRJNA946291
SAMN37499067	Yojoa_06_2019_Q_hypo_A_bin.97	Yojoa_06_2019_Q_hypo_A_bin.97	Nanoarchaeota archaeon	2026764	PRJNA946291
SAMN37499068	Yojoa_06_2019_Q_hypo_AN_bin.100	Yojoa_06_2019_Q_hypo_AN_bin.100	Bacteroidota bacterium	1898104	PRJNA946291
SAMN37499069	Yojoa_06_2019_Q_hypo_AN_bin.102	Yojoa_06_2019_Q_hypo_AN_bin.102	Candidatus Diapherotrites archaeon	2026736	PRJNA946291
SAMN37499070	Yojoa_06_2019_Q_hypo_AN_bin.15	Yojoa_06_2019_Q_hypo_AN_bin.15	Candidatus Parcubacteria bacterium	2762014	PRJNA946291
SAMN37499071	Yojoa_06_2019_Q_hypo_AN_bin.42	Yojoa_06_2019_Q_hypo_AN_bin.42	Chloroflexota bacterium	2026724	PRJNA946291
SAMN37499072	Yojoa_06_2019_Q_hypo_AN_bin.50	Yojoa_06_2019_Q_hypo_AN_bin.50	Verrucomicrobiota bacterium	2026799	PRJNA946291
SAMN37499073	Yojoa_06_2019_Q_hypo_AN_bin.55	Yojoa_06_2019_Q_hypo_AN_bin.55	Candidatus Diapherotrites archaeon	2026736	PRJNA946291
SAMN37499074	Yojoa_06_2019_Q_hypo_AN_bin.56	Yojoa_06_2019_Q_hypo_AN_bin.56	Verrucomicrobiota bacterium	2026799	PRJNA946291
SAMN37499075	Yojoa_06_2019_Q_hypo_AN_bin.72	Yojoa_06_2019_Q_hypo_AN_bin.72	Verrucomicrobiota bacterium	2026799	PRJNA946291

SAMN37499076	Yojoa_06_2019_Q_hypo_AN_bin.92	Yojoa_06_2019_Q_hypo_AN_bin.92	Bacteroidota bacterium	1898104	PRJNA946291
SAMN37499077	Yojoa_06_2019_Q_hypo_AN_bin.94	Yojoa_06_2019_Q_hypo_AN_bin.94	Candidatus Parcubacteria bacterium	2762014	PRJNA946291
SAMN37499078	Yojoa_06_2019_Q_hypo_AN_bin.96	Yojoa_06_2019_Q_hypo_AN_bin.96	Candidatus Omnitrophota bacterium	2035772	PRJNA946291
SAMN37499079	Yojoa_06_2019_Q_hypo_AN_bin.97	Yojoa_06_2019_Q_hypo_AN_bin.97	Bacteroidota bacterium	1898104	PRJNA946291
SAMN37499080	Yojoa_01_2020_B_meta_A_bin.39	Yojoa_01_2020_B_meta_A_bin.39	Planctomycetota bacterium	2026780	PRJNA946291
SAMN37499081	Yojoa_01_2020_B_meta_A_bin.41	Yojoa_01_2020_B_meta_A_bin.41	Pseudomonadota bacterium	1977087	PRJNA946291
SAMN37499082	Yojoa_01_2020_B_meta_A_bin.73	Yojoa_01_2020_B_meta_A_bin.73	Pseudomonadota bacterium	1977087	PRJNA946291
SAMN37499083	Yojoa_01_2020_B_meta_AN_bin.21	Yojoa_01_2020_B_meta_AN_bin.21	Actinomycetota bacterium	2900548	PRJNA946291
SAMN37499084	Yojoa_06_2019_E_epi_A_bin.43	Yojoa_06_2019_E_epi_A_bin.43	Pseudomonadota bacterium	1977087	PRJNA946291
SAMN37499085	Yojoa_06_2019_E_epi_A_bin.51	Yojoa_06_2019_E_epi_A_bin.51	Verrucomicrobiota bacterium	2026799	PRJNA946291
SAMN37499086	Yojoa_06_2019_E_epi_A_bin.70	Yojoa_06_2019_E_epi_A_bin.70	Bacteroidota bacterium	1898104	PRJNA946291
SAMN37499087	Yojoa_06_2019_E_epi_A_bin.99	Yojoa_06_2019_E_epi_A_bin.99	Cyanobacteriota bacterium	2847862	PRJNA946291
SAMN37499088	Yojoa_06_2019_E_epi_AN_bin.11	Yojoa_06_2019_E_epi_AN_bin.11	Chlamydiota bacterium	2081524	PRJNA946291
SAMN37499089	Yojoa_06_2019_E_epi_AN_bin.5	Yojoa_06_2019_E_epi_AN_bin.5	Cyanobacteriota bacterium	2847862	PRJNA946291
SAMN37499090	Yojoa_06_2019_L_epi_A_bin.123	Yojoa_06_2019_L_epi_A_bin.123	Cyanobacteriota bacterium	2847862	PRJNA946291
SAMN37499091	Yojoa_06_2019_L_epi_A_bin.25	Yojoa_06_2019_L_epi_A_bin.25	Pseudomonadota bacterium	1977087	PRJNA946291
SAMN37499092	Yojoa_06_2019_L_epi_A_bin.30	Yojoa_06_2019_L_epi_A_bin.30	Bacteroidota bacterium	1898104	PRJNA946291
SAMN37499093	Yojoa_06_2019_L_epi_A_bin.54	Yojoa_06_2019_L_epi_A_bin.54	Bacteroidota bacterium	1898104	PRJNA946291
SAMN37499094	Yojoa_06_2019_L_epi_A_bin.61	Yojoa_06_2019_L_epi_A_bin.61	Candidatus Parcubacteria bacterium	2762014	PRJNA946291
SAMN37499095	Yojoa_06_2019_L_epi_A_bin.68	Yojoa_06_2019_L_epi_A_bin.68	Pseudomonadota bacterium	1977087	PRJNA946291
SAMN37499096	Yojoa_06_2019_L_epi_A_bin.70	Yojoa_06_2019_L_epi_A_bin.70	Pseudomonadota bacterium	1977087	PRJNA946291
SAMN37499097	Yojoa_06_2019_L_epi_A_bin.75	Yojoa_06_2019_L_epi_A_bin.75	Planctomycetota bacterium	2026780	PRJNA946291
SAMN37499098	Yojoa_06_2019_L_epi_A_bin.87	Yojoa_06_2019_L_epi_A_bin.87	Bacteroidota bacterium	1898104	PRJNA946291
SAMN37499099	Yojoa_06_2019_L_epi_AN_bin.40	Yojoa_06_2019_L_epi_AN_bin.40	Bacteroidota bacterium	1898104	PRJNA946291
SAMN37499100	Yojoa_06_2019_L_epi_AN_bin.50	Yojoa_06_2019_L_epi_AN_bin.50	Actinomycetota bacterium	2900548	PRJNA946291
SAMN37499101	Yojoa_06_2019_L_epi_AN_bin.54	Yojoa_06_2019_L_epi_AN_bin.54	Bacteroidota bacterium	1898104	PRJNA946291
SAMN37499102	Yojoa_06_2019_L_epi_AN_bin.60	Yojoa_06_2019_L_epi_AN_bin.60	Pseudomonadota bacterium	1977087	PRJNA946291
SAMN37499103	Yojoa_06_2019_Q_epi_A_bin.15	Yojoa_06_2019_Q_epi_A_bin.15	Pseudomonadota bacterium	1977087	PRJNA946291
SAMN37499104	Yojoa_06_2019_Q_epi_A_bin.16	Yojoa_06_2019_Q_epi_A_bin.16	Actinomycetota bacterium	2900548	PRJNA946291
SAMN37499105	Yojoa_06_2019_Q_epi_A_bin.21	Yojoa_06_2019_Q_epi_A_bin.21	Cyanobacteriota bacterium	2847862	PRJNA946291
SAMN37499106	Yojoa_06_2019_Q_epi_A_bin.22	Yojoa_06_2019_Q_epi_A_bin.22	Pseudomonadota bacterium	1977087	PRJNA946291
SAMN37499107	Yojoa_06_2019_Q_epi_A_bin.24	Yojoa_06_2019_Q_epi_A_bin.24	Planctomycetota bacterium	2026780	PRJNA946291
SAMN37499108	Yojoa_06_2019_Q_epi_A_bin.35	Yojoa_06_2019_Q_epi_A_bin.35	Pseudomonadota bacterium	1977087	PRJNA946291
SAMN37499109	Yojoa_06_2019_Q_epi_A_bin.44	Yojoa_06_2019_Q_epi_A_bin.44	Actinomycetota bacterium	2900548	PRJNA946291
SAMN37499110	Yojoa_06_2019_Q_epi_A_bin.53	Yojoa_06_2019_Q_epi_A_bin.53	Gemmatimonadota bacterium	2026742	PRJNA946291
SAMN37499111	Yojoa_06_2019_Q_epi_A_bin.56	Yojoa_06_2019_Q_epi_A_bin.56	Planctomycetota bacterium	2026780	PRJNA946291

SAMN37499112	Yojoa_06_2019_Q_epi_A_bin.65	Yojoa_06_2019_Q_epi_A_bin.65	Actinomycetota bacterium	2900548	PRJNA946291
SAMN37499113	Yojoa_06_2019_Q_epi_A_bin.8	Yojoa_06_2019_Q_epi_A_bin.8	Pseudomonadota bacterium	1977087	PRJNA946291
SAMN37499114	Yojoa_06_2019_Q_epi_A_bin.85	Yojoa_06_2019_Q_epi_A_bin.85	Pseudomonadota bacterium	1977087	PRJNA946291
SAMN37499115	Yojoa_06_2019_Q_epi_A_bin.87	Yojoa_06_2019_Q_epi_A_bin.87	Pseudomonadota bacterium	1977087	PRJNA946291
SAMN37499116	Yojoa_06_2019_Q_epi_A_bin.90	Yojoa_06_2019_Q_epi_A_bin.90	Pseudomonadota bacterium	1977087	PRJNA946291
SAMN37499117	Yojoa_06_2019_Q_epi_AN_bin.51	Yojoa_06_2019_Q_epi_AN_bin.51	Actinomycetota bacterium	2900548	PRJNA946291
SAMN37499118	Yojoa_01_2020_E_meta_A_bin.117	Yojoa_01_2020_E_meta_A_bin.117	Pseudomonadota bacterium	1977087	PRJNA946291
SAMN37499119	Yojoa_01_2020_E_meta_A_bin.3	Yojoa_01_2020_E_meta_A_bin.3	Pseudomonadota bacterium	1977087	PRJNA946291
SAMN37499120	Yojoa_01_2020_E_meta_A_bin.46	Yojoa_01_2020_E_meta_A_bin.46	Pseudomonadota bacterium	1977087	PRJNA946291
SAMN37499121	Yojoa_01_2020_E_meta_A_bin.49	Yojoa_01_2020_E_meta_A_bin.49	Actinomycetota bacterium	2900548	PRJNA946291
SAMN37499122	Yojoa_01_2020_E_meta_A_bin.54	Yojoa_01_2020_E_meta_A_bin.54	Pseudomonadota bacterium	1977087	PRJNA946291
SAMN37499123	Yojoa_01_2020_E_meta_A_bin.60	Yojoa_01_2020_E_meta_A_bin.60	Verrucomicrobiota bacterium	2026799	PRJNA946291
SAMN37499124	Yojoa_01_2020_E_meta_A_bin.62	Yojoa_01_2020_E_meta_A_bin.62	Planctomycetota bacterium	2026780	PRJNA946291
SAMN37499125	Yojoa_01_2020_E_meta_A_bin.77	Yojoa_01_2020_E_meta_A_bin.77	Pseudomonadota bacterium	1977087	PRJNA946291
SAMN37499126	Yojoa_01_2020_E_epi_A_bin.48	Yojoa_01_2020_E_epi_A_bin.48	Pseudomonadota bacterium	1977087	PRJNA946291
SAMN37499127	Yojoa_01_2020_E_epi_A_bin.52	Yojoa_01_2020_E_epi_A_bin.52	Pseudomonadota bacterium	1977087	PRJNA946291
SAMN37499128	Yojoa_01_2020_E_epi_AN_bin.39	Yojoa_01_2020_E_epi_AN_bin.39	Chlamydiota bacterium	2081524	PRJNA946291
SAMN37499129	Yojoa_01_2020_E_hypo_A_bin.104	Yojoa_01_2020_E_hypo_A_bin.104	Actinomycetota bacterium	2900548	PRJNA946291
SAMN37499130	Yojoa_01_2020_E_hypo_A_bin.12	Yojoa_01_2020_E_hypo_A_bin.12	Planctomycetota bacterium	2026780	PRJNA946291
SAMN37499131	Yojoa_01_2020_E_hypo_A_bin.21	Yojoa_01_2020_E_hypo_A_bin.21	Actinomycetota bacterium	2900548	PRJNA946291
SAMN37499132	Yojoa_01_2020_E_hypo_A_bin.41	Yojoa_01_2020_E_hypo_A_bin.41	Bacteroidota bacterium	1898104	PRJNA946291
SAMN37499133	Yojoa_01_2020_E_hypo_A_bin.43	Yojoa_01_2020_E_hypo_A_bin.43	Chloroflexota bacterium	2026724	PRJNA946291
SAMN37499134	Yojoa_01_2020_E_hypo_A_bin.47	Yojoa_01_2020_E_hypo_A_bin.47	Cyanobacteriota bacterium	2847862	PRJNA946291
SAMN37499135	Yojoa_01_2020_E_hypo_A_bin.55	Yojoa_01_2020_E_hypo_A_bin.55	Actinomycetota bacterium	2900548	PRJNA946291
SAMN37499136	Yojoa_01_2020_E_hypo_A_bin.69	Yojoa_01_2020_E_hypo_A_bin.69	Actinomycetota bacterium	2900548	PRJNA946291
SAMN37499137	Yojoa_01_2020_E_hypo_AN_bin.4	Yojoa_01_2020_E_hypo_AN_bin.4	Cyanobacteriota bacterium	2847862	PRJNA946291
SAMN37499138	Yojoa_01_2020_B_hypo_A_bin.40	Yojoa_01_2020_B_hypo_A_bin.40	Actinomycetota bacterium	2900548	PRJNA946291
SAMN37499139	Yojoa_01_2020_B_epi_A_bin.20	Yojoa_01_2020_B_epi_A_bin.20	Pseudomonadota bacterium	1977087	PRJNA946291
SAMN37499140	Yojoa_01_2020_R_hypo_A_bin.103	Yojoa_01_2020_R_hypo_A_bin.103	Pseudomonadota bacterium	1977087	PRJNA946291
SAMN37499141	Yojoa_01_2020_R_hypo_A_bin.5	Yojoa_01_2020_R_hypo_A_bin.5	Pseudomonadota bacterium	1977087	PRJNA946291
SAMN37499142	Yojoa_01_2020_R_meta_A_bin.31	Yojoa_01_2020_R_meta_A_bin.31	Chlamydiota bacterium	2081524	PRJNA946291
SAMN37499143	Yojoa_01_2020_R_meta_A_bin.46	Yojoa_01_2020_R_meta_A_bin.46	Actinomycetota bacterium	2900548	PRJNA946291
SAMN37499144	Yojoa_01_2020_R_meta_A_bin.55	Yojoa_01_2020_R_meta_A_bin.55	Chlamydiota bacterium	2081524	PRJNA946291
SAMN37499145	Yojoa_01_2020_R_meta_A_bin.80	Yojoa_01_2020_R_meta_A_bin.80	Chlamydiota bacterium	2081524	PRJNA946291
SAMN37499146	Yojoa_01_2022_B_epi_A_bin.10	Yojoa_01_2022_B_epi_A_bin.10	Actinomycetota bacterium	2900548	PRJNA946291
SAMN37499147	Yojoa_01_2022_B_epi_A_bin.108	Yojoa_01_2022_B_epi_A_bin.108	Pseudomonadota bacterium	1977087	PRJNA946291

SAMN37499148	Yojoa_01_2022_B_epi_A_bin.116	Yojoa_01_2022_B_epi_A_bin.116	Pseudomonadota bacterium	1977087	PRJNA946291
SAMN37499149	Yojoa_01_2022_B_epi_A_bin.133	Yojoa_01_2022_B_epi_A_bin.133	Actinomycetota bacterium	2900548	PRJNA946291
SAMN37499150	Yojoa_01_2022_B_epi_A_bin.139	Yojoa_01_2022_B_epi_A_bin.139	Bacteroidota bacterium	1898104	PRJNA946291
SAMN37499151	Yojoa_01_2022_B_epi_A_bin.145	Yojoa_01_2022_B_epi_A_bin.145	Bacteroidota bacterium	1898104	PRJNA946291
SAMN37499152	Yojoa_01_2022_B_epi_A_bin.147	Yojoa_01_2022_B_epi_A_bin.147	Verrucomicrobiota bacterium	2026799	PRJNA946291
SAMN37499153	Yojoa_01_2022_B_epi_A_bin.154	Yojoa_01_2022_B_epi_A_bin.154	Pseudomonadota bacterium	1977087	PRJNA946291
SAMN37499154	Yojoa_01_2022_B_epi_A_bin.161	Yojoa_01_2022_B_epi_A_bin.161	Actinomycetota bacterium	2900548	PRJNA946291
SAMN37499155	Yojoa_01_2022_B_epi_A_bin.164	Yojoa_01_2022_B_epi_A_bin.164	Pseudomonadota bacterium	1977087	PRJNA946291
SAMN37499156	Yojoa_01_2022_B_epi_A_bin.173	Yojoa_01_2022_B_epi_A_bin.173	Planctomycetota bacterium	2026780	PRJNA946291
SAMN37499157	Yojoa_01_2022_B_epi_A_bin.182	Yojoa_01_2022_B_epi_A_bin.182	Bacteroidota bacterium	1898104	PRJNA946291
SAMN37499158	Yojoa_01_2022_B_epi_A_bin.2	Yojoa_01_2022_B_epi_A_bin.2	Pseudomonadota bacterium	1977087	PRJNA946291
SAMN37499159	Yojoa_01_2022_B_epi_A_bin.54	Yojoa_01_2022_B_epi_A_bin.54	Pseudomonadota bacterium	1977087	PRJNA946291
SAMN37499160	Yojoa_01_2022_B_epi_A_bin.62	Yojoa_01_2022_B_epi_A_bin.62	Bacteroidota bacterium	1898104	PRJNA946291
SAMN37499161	Yojoa_01_2022_B_epi_A_bin.7	Yojoa_01_2022_B_epi_A_bin.7	Bacteroidota bacterium	1898104	PRJNA946291
SAMN37499162	Yojoa_01_2022_B_epi_A_bin.71	Yojoa_01_2022_B_epi_A_bin.71	Verrucomicrobiota bacterium	2026799	PRJNA946291
SAMN37499163	Yojoa_01_2022_B_epi_A_bin.93	Yojoa_01_2022_B_epi_A_bin.93	Actinomycetota bacterium	2900548	PRJNA946291
SAMN37499164	Yojoa_01_2022_B_epi_K_bin.126	Yojoa_01_2022_B_epi_K_bin.126	Pseudomonadota bacterium	1977087	PRJNA946291
SAMN37499165	Yojoa_01_2022_B_epi_K_bin.21	Yojoa_01_2022_B_epi_K_bin.21	Bacteroidota bacterium	1898104	PRJNA946291
SAMN37499166	Yojoa_01_2022_B_epi_K_bin.47	Yojoa_01_2022_B_epi_K_bin.47	Planctomycetota bacterium	2026780	PRJNA946291
SAMN37499167	Yojoa_01_2022_B_epi_K_bin.51	Yojoa_01_2022_B_epi_K_bin.51	Planctomycetota bacterium	2026780	PRJNA946291
SAMN37499168	Yojoa_01_2022_B_epi_K_bin.6	Yojoa_01_2022_B_epi_K_bin.6	Pseudomonadota bacterium	1977087	PRJNA946291
SAMN37499169	Yojoa_01_2022_B_epi_K_bin.71	Yojoa_01_2022_B_epi_K_bin.71	Bacteroidota bacterium	1898104	PRJNA946291
SAMN37499170	Yojoa_01_2022_B_epi_K_bin.72	Yojoa_01_2022_B_epi_K_bin.72	Actinomycetota bacterium	2900548	PRJNA946291
SAMN37499171	Yojoa_01_2022_B_hypo_A_bin.103	Yojoa_01_2022_B_hypo_A_bin.103	Acidobacteriota bacterium	1978231	PRJNA946291
SAMN37499172	Yojoa_01_2022_B_hypo_A_bin.105	Yojoa_01_2022_B_hypo_A_bin.105	Bacteroidota bacterium	1898104	PRJNA946291
SAMN37499173	Yojoa_01_2022_B_hypo_A_bin.108	Yojoa_01_2022_B_hypo_A_bin.108	Bacteroidota bacterium	1898104	PRJNA946291
SAMN37499174	Yojoa_01_2022_B_hypo_A_bin.110	Yojoa_01_2022_B_hypo_A_bin.110	Verrucomicrobiota bacterium	2026799	PRJNA946291
SAMN37499175	Yojoa_01_2022_B_hypo_A_bin.117	Yojoa_01_2022_B_hypo_A_bin.117	Bacteroidota bacterium	1898104	PRJNA946291
SAMN37499176	Yojoa_01_2022_B_hypo_A_bin.128	Yojoa_01_2022_B_hypo_A_bin.128	Actinomycetota bacterium	2900548	PRJNA946291
SAMN37499177	Yojoa_01_2022_B_hypo_A_bin.13	Yojoa_01_2022_B_hypo_A_bin.13	Planctomycetota bacterium	2026780	PRJNA946291
SAMN37499178	Yojoa_01_2022_B_hypo_A_bin.15	Yojoa_01_2022_B_hypo_A_bin.15	Cyanobacteriota bacterium	2847862	PRJNA946291
SAMN37499179	Yojoa_01_2022_B_hypo_A_bin.153	Yojoa_01_2022_B_hypo_A_bin.153	Bacteroidota bacterium	1898104	PRJNA946291
SAMN37499180	Yojoa_01_2022_B_hypo_A_bin.158	Yojoa_01_2022_B_hypo_A_bin.158	Planctomycetota bacterium	2026780	PRJNA946291
SAMN37499181	Yojoa_01_2022_B_hypo_A_bin.195	Yojoa_01_2022_B_hypo_A_bin.195	Pseudomonadota bacterium	1977087	PRJNA946291
SAMN37499182	Yojoa_01_2022_B_hypo_A_bin.41	Yojoa_01_2022_B_hypo_A_bin.41	Verrucomicrobiota bacterium	2026799	PRJNA946291
SAMN37499183	Yojoa_01_2022_B_hypo_A_bin.47	Yojoa_01_2022_B_hypo_A_bin.47	Planctomycetota bacterium	2026780	PRJNA946291

SAMN37499184	Yojoa_01_2022_B_hypo_A_bin.50	Yojoa_01_2022_B_hypo_A_bin.50	Planctomycetota bacterium	2026780	PRJNA946291
SAMN37499185	Yojoa_01_2022_B_hypo_A_bin.59	Yojoa_01_2022_B_hypo_A_bin.59	Verrucomicrobiota bacterium	2026799	PRJNA946291
SAMN37499186	Yojoa_01_2022_B_hypo_A_bin.68	Yojoa_01_2022_B_hypo_A_bin.68	Actinomycetota bacterium	2900548	PRJNA946291
SAMN37499187	Yojoa_01_2022_B_hypo_A_bin.80	Yojoa_01_2022_B_hypo_A_bin.80	Actinomycetota bacterium	2900548	PRJNA946291
SAMN37499188	Yojoa_01_2022_B_hypo_A_bin.83	Yojoa_01_2022_B_hypo_A_bin.83	Planctomycetota bacterium	2026780	PRJNA946291
SAMN37499189	Yojoa_01_2022_B_hypo_A_bin.90	Yojoa_01_2022_B_hypo_A_bin.90	Actinomycetota bacterium	2900548	PRJNA946291
SAMN37499190	Yojoa_01_2022_B_hypo_A_bin.96	Yojoa_01_2022_B_hypo_A_bin.96	Verrucomicrobiota bacterium	2026799	PRJNA946291
SAMN37499191	Yojoa_01_2022_B_hypo_K_bin.13	Yojoa_01_2022_B_hypo_K_bin.13	Verrucomicrobiota bacterium	2026799	PRJNA946291
SAMN37499192	Yojoa_01_2022_B_hypo_K_bin.131	Yojoa_01_2022_B_hypo_K_bin.131	Bacteroidota bacterium	1898104	PRJNA946291
SAMN37499193	Yojoa_01_2022_B_hypo_K_bin.14	Yojoa_01_2022_B_hypo_K_bin.14	Actinomycetota bacterium	2900548	PRJNA946291
SAMN37499194	Yojoa_01_2022_B_hypo_K_bin.35	Yojoa_01_2022_B_hypo_K_bin.35	Pseudomonadota bacterium	1977087	PRJNA946291
SAMN37499195	Yojoa_01_2022_B_hypo_K_bin.56	Yojoa_01_2022_B_hypo_K_bin.56	Planctomycetota bacterium	2026780	PRJNA946291
SAMN37499196	Yojoa_01_2022_E_epi_A_bin.107	Yojoa_01_2022_E_epi_A_bin.107	Verrucomicrobiota bacterium	2026799	PRJNA946291
SAMN37499197	Yojoa_01_2022_E_epi_A_bin.113	Yojoa_01_2022_E_epi_A_bin.113	Actinomycetota bacterium	2900548	PRJNA946291
SAMN37499198	Yojoa_01_2022_E_epi_A_bin.114	Yojoa_01_2022_E_epi_A_bin.114	Bacteroidota bacterium	1898104	PRJNA946291
SAMN37499199	Yojoa_01_2022_E_epi_A_bin.124	Yojoa_01_2022_E_epi_A_bin.124	Bacteroidota bacterium	1898104	PRJNA946291
SAMN37499200	Yojoa_01_2022_E_epi_A_bin.132	Yojoa_01_2022_E_epi_A_bin.132	Pseudomonadota bacterium	1977087	PRJNA946291
SAMN37499201	Yojoa_01_2022_E_epi_A_bin.139	Yojoa_01_2022_E_epi_A_bin.139	Pseudomonadota bacterium	1977087	PRJNA946291
SAMN37499202	Yojoa_01_2022_E_epi_A_bin.143	Yojoa_01_2022_E_epi_A_bin.143	Bacteroidota bacterium	1898104	PRJNA946291
SAMN37499203	Yojoa_01_2022_E_epi_A_bin.144	Yojoa_01_2022_E_epi_A_bin.144	Pseudomonadota bacterium	1977087	PRJNA946291
SAMN37499204	Yojoa_01_2022_E_epi_A_bin.164	Yojoa_01_2022_E_epi_A_bin.164	Bacteroidota bacterium	1898104	PRJNA946291
SAMN37499205	Yojoa_01_2022_E_epi_A_bin.173	Yojoa_01_2022_E_epi_A_bin.173	Pseudomonadota bacterium	1977087	PRJNA946291
SAMN37499206	Yojoa_01_2022_E_epi_A_bin.174	Yojoa_01_2022_E_epi_A_bin.174	Chloroflexota bacterium	2026724	PRJNA946291
SAMN37499207	Yojoa_01_2022_E_epi_A_bin.21	Yojoa_01_2022_E_epi_A_bin.21	Bacteroidota bacterium	1898104	PRJNA946291
SAMN37499208	Yojoa_01_2022_E_epi_A_bin.217	Yojoa_01_2022_E_epi_A_bin.217	Acidobacteriota bacterium	1978231	PRJNA946291
SAMN37499209	Yojoa_01_2022_E_epi_A_bin.27	Yojoa_01_2022_E_epi_A_bin.27	Actinomycetota bacterium	2900548	PRJNA946291
SAMN37499210	Yojoa_01_2022_E_epi_A_bin.3	Yojoa_01_2022_E_epi_A_bin.3	Bacteroidota bacterium	1898104	PRJNA946291
SAMN37499211	Yojoa_01_2022_E_epi_A_bin.62	Yojoa_01_2022_E_epi_A_bin.62	Planctomycetota bacterium	2026780	PRJNA946291
SAMN37499212	Yojoa_01_2022_E_epi_A_bin.64	Yojoa_01_2022_E_epi_A_bin.64	Planctomycetota bacterium	2026780	PRJNA946291
SAMN37499213	Yojoa_01_2022_E_epi_A_bin.66	Yojoa_01_2022_E_epi_A_bin.66	Bacteroidota bacterium	1898104	PRJNA946291
SAMN37499214	Yojoa_01_2022_E_epi_A_bin.68	Yojoa_01_2022_E_epi_A_bin.68	Pseudomonadota bacterium	1977087	PRJNA946291
SAMN37499215	Yojoa_01_2022_E_epi_A_bin.77	Yojoa_01_2022_E_epi_A_bin.77	Planctomycetota bacterium	2026780	PRJNA946291
SAMN37499216	Yojoa_01_2022_E_epi_A_bin.81	Yojoa_01_2022_E_epi_A_bin.81	Planctomycetota bacterium	2026780	PRJNA946291
SAMN37499217	Yojoa_01_2022_E_epi_A_bin.82	Yojoa_01_2022_E_epi_A_bin.82	Bacteroidota bacterium	1898104	PRJNA946291
SAMN37499218	Yojoa_01_2022_E_epi_K_bin.138	Yojoa_01_2022_E_epi_K_bin.138	Actinomycetota bacterium	2900548	PRJNA946291
SAMN37499219	Yojoa_01_2022_E_epi_K_bin.16	Yojoa_01_2022_E_epi_K_bin.16	Pseudomonadota bacterium	1977087	PRJNA946291

SAMN37499220	Yojoa_01_2022_E_epi_K_bin.35	Yojoa_01_2022_E_epi_K_bin.35	Pseudomonadota bacterium	1977087	PRJNA946291
SAMN37499221	Yojoa_01_2022_E_epi_K_bin.6	Yojoa_01_2022_E_epi_K_bin.6	Actinomycetota bacterium	2900548	PRJNA946291
SAMN37499222	Yojoa_01_2022_E_epi_K_bin.74	Yojoa_01_2022_E_epi_K_bin.74	Pseudomonadota bacterium	1977087	PRJNA946291
SAMN37499223	Yojoa_01_2022_E_epi_K_bin.76	Yojoa_01_2022_E_epi_K_bin.76	Pseudomonadota bacterium	1977087	PRJNA946291
SAMN37499224	Yojoa_01_2022_E_hypo_A_bin.1	Yojoa_01_2022_E_hypo_A_bin.1	Pseudomonadota bacterium	1977087	PRJNA946291
SAMN37499225	Yojoa_01_2022_E_hypo_A_bin.113	Yojoa_01_2022_E_hypo_A_bin.113	Bacteroidota bacterium	1898104	PRJNA946291
SAMN37499226	Yojoa_01_2022_E_hypo_A_bin.116	Yojoa_01_2022_E_hypo_A_bin.116	Pseudomonadota bacterium	1977087	PRJNA946291
SAMN37499227	Yojoa_01_2022_E_hypo_A_bin.121	Yojoa_01_2022_E_hypo_A_bin.121	Bacteroidota bacterium	1898104	PRJNA946291
SAMN37499228	Yojoa_01_2022_E_hypo_A_bin.126	Yojoa_01_2022_E_hypo_A_bin.126	Actinomycetota bacterium	2900548	PRJNA946291
SAMN37499229	Yojoa_01_2022_E_hypo_A_bin.155	Yojoa_01_2022_E_hypo_A_bin.155	Cyanobacteriota bacterium	2847862	PRJNA946291
SAMN37499230	Yojoa_01_2022_E_hypo_A_bin.164	Yojoa_01_2022_E_hypo_A_bin.164	Bacillota bacterium	1879010	PRJNA946291
SAMN37499231	Yojoa_01_2022_E_hypo_A_bin.174	Yojoa_01_2022_E_hypo_A_bin.174	Pseudomonadota bacterium	1977087	PRJNA946291
SAMN37499232	Yojoa_01_2022_E_hypo_A_bin.24	Yojoa_01_2022_E_hypo_A_bin.24	Pseudomonadota bacterium	1977087	PRJNA946291
SAMN37499233	Yojoa_01_2022_E_hypo_A_bin.31	Yojoa_01_2022_E_hypo_A_bin.31	Actinomycetota bacterium	2900548	PRJNA946291
SAMN37499234	Yojoa_01_2022_E_hypo_A_bin.5	Yojoa_01_2022_E_hypo_A_bin.5	Planctomycetota bacterium	2026780	PRJNA946291
SAMN37499235	Yojoa_01_2022_E_hypo_A_bin.59	Yojoa_01_2022_E_hypo_A_bin.59	Pseudomonadota bacterium	1977087	PRJNA946291
SAMN37499236	Yojoa_01_2022_E_hypo_A_bin.63	Yojoa_01_2022_E_hypo_A_bin.63	Pseudomonadota bacterium	1977087	PRJNA946291
SAMN37499237	Yojoa_01_2022_E_hypo_A_bin.65	Yojoa_01_2022_E_hypo_A_bin.65	Pseudomonadota bacterium	1977087	PRJNA946291
SAMN37499238	Yojoa_01_2022_E_hypo_A_bin.66	Yojoa_01_2022_E_hypo_A_bin.66	Cyanobacteriota bacterium	2847862	PRJNA946291
SAMN37499239	Yojoa_01_2022_E_hypo_A_bin.69	Yojoa_01_2022_E_hypo_A_bin.69	Chloroflexota bacterium	2026724	PRJNA946291
SAMN37499240	Yojoa_01_2022_E_hypo_A_bin.73	Yojoa_01_2022_E_hypo_A_bin.73	Cyanobacteriota bacterium	2847862	PRJNA946291
SAMN37499241	Yojoa_01_2022_E_hypo_A_bin.78	Yojoa_01_2022_E_hypo_A_bin.78	Planctomycetota bacterium	2026780	PRJNA946291
SAMN37499242	Yojoa_01_2022_E_hypo_A_bin.94	Yojoa_01_2022_E_hypo_A_bin.94	Planctomycetota bacterium	2026780	PRJNA946291
SAMN37499243	Yojoa_01_2022_E_hypo_K_bin.106	Yojoa_01_2022_E_hypo_K_bin.106	Bacteroidota bacterium	1898104	PRJNA946291
SAMN37499244	Yojoa_01_2022_E_hypo_K_bin.110	Yojoa_01_2022_E_hypo_K_bin.110	Planctomycetota bacterium	2026780	PRJNA946291
SAMN37499245	Yojoa_01_2022_E_hypo_K_bin.19	Yojoa_01_2022_E_hypo_K_bin.19	Pseudomonadota bacterium	1977087	PRJNA946291
SAMN37499246	Yojoa_01_2022_E_hypo_K_bin.44	Yojoa_01_2022_E_hypo_K_bin.44	Planctomycetota bacterium	2026780	PRJNA946291
SAMN37499247	Yojoa_01_2022_E_hypo_K_bin.86	Yojoa_01_2022_E_hypo_K_bin.86	Pseudomonadota bacterium	1977087	PRJNA946291
SAMN37499248	Yojoa_01_2022_E_hypo_K_bin.95	Yojoa_01_2022_E_hypo_K_bin.95	Bacteroidota bacterium	1898104	PRJNA946291
SAMN37499249	Yojoa_01_2022_R_epi_A_bin.116	Yojoa_01_2022_R_epi_A_bin.116	Planctomycetota bacterium	2026780	PRJNA946291
SAMN37499250	Yojoa_01_2022_R_epi_A_bin.121	Yojoa_01_2022_R_epi_A_bin.121	Actinomycetota bacterium	2900548	PRJNA946291
SAMN37499251	Yojoa_01_2022_R_epi_A_bin.126	Yojoa_01_2022_R_epi_A_bin.126	Actinomycetota bacterium	2900548	PRJNA946291
SAMN37499252	Yojoa_01_2022_R_epi_A_bin.134	Yojoa_01_2022_R_epi_A_bin.134	Actinomycetota bacterium	2900548	PRJNA946291
SAMN37499253	Yojoa_01_2022_R_epi_A_bin.146	Yojoa_01_2022_R_epi_A_bin.146	Verrucomicrobiota bacterium	2026799	PRJNA946291
SAMN37499254	Yojoa_01_2022_R_epi_A_bin.158	Yojoa_01_2022_R_epi_A_bin.158	Pseudomonadota bacterium	1977087	PRJNA946291
SAMN37499255	Yojoa_01_2022_R_epi_A_bin.163	Yojoa_01_2022_R_epi_A_bin.163	Verrucomicrobiota bacterium	2026799	PRJNA946291

SAMN37499256	Yojoa_01_2022_R_epi_A_bin.168	Yojoa_01_2022_R_epi_A_bin.168	Actinomycetota bacterium	2900548	PRJNA946291
SAMN37499257	Yojoa_01_2022_R_epi_A_bin.170	Yojoa_01_2022_R_epi_A_bin.170	Chlamydiota bacterium	2081524	PRJNA946291
SAMN37499258	Yojoa_01_2022_R_epi_A_bin.173	Yojoa_01_2022_R_epi_A_bin.173	Pseudomonadota bacterium	1977087	PRJNA946291
SAMN37499259	Yojoa_01_2022_R_epi_A_bin.179	Yojoa_01_2022_R_epi_A_bin.179	Planctomycetota bacterium	2026780	PRJNA946291
SAMN37499260	Yojoa_01_2022_R_epi_A_bin.183	Yojoa_01_2022_R_epi_A_bin.183	Pseudomonadota bacterium	1977087	PRJNA946291
SAMN37499261	Yojoa_01_2022_R_epi_A_bin.27	Yojoa_01_2022_R_epi_A_bin.27	Verrucomicrobiota bacterium	2026799	PRJNA946291
SAMN37499262	Yojoa_01_2022_R_epi_A_bin.31	Yojoa_01_2022_R_epi_A_bin.31	Bacteroidota bacterium	1898104	PRJNA946291
SAMN37499263	Yojoa_01_2022_R_epi_A_bin.40	Yojoa_01_2022_R_epi_A_bin.40	Bacteroidota bacterium	1898104	PRJNA946291
SAMN37499264	Yojoa_01_2022_R_epi_A_bin.55	Yojoa_01_2022_R_epi_A_bin.55	Chloroflexota bacterium	2026724	PRJNA946291
SAMN37499265	Yojoa_01_2022_R_epi_A_bin.6	Yojoa_01_2022_R_epi_A_bin.6	Actinomycetota bacterium	2900548	PRJNA946291
SAMN37499266	Yojoa_01_2022_R_epi_A_bin.66	Yojoa_01_2022_R_epi_A_bin.66	Pseudomonadota bacterium	1977087	PRJNA946291
SAMN37499267	Yojoa_01_2022_R_epi_A_bin.68	Yojoa_01_2022_R_epi_A_bin.68	Actinomycetota bacterium	2900548	PRJNA946291
SAMN37499268	Yojoa_01_2022_R_epi_A_bin.75	Yojoa_01_2022_R_epi_A_bin.75	Cyanobacteriota bacterium	2847862	PRJNA946291
SAMN37499269	Yojoa_01_2022_R_epi_A_bin.90	Yojoa_01_2022_R_epi_A_bin.90	Bacteroidota bacterium	1898104	PRJNA946291
SAMN37499270	Yojoa_01_2022_R_epi_A_bin.96	Yojoa_01_2022_R_epi_A_bin.96	Chloroflexota bacterium	2026724	PRJNA946291
SAMN37499271	Yojoa_01_2022_R_epi_K_bin.113	Yojoa_01_2022_R_epi_K_bin.113	Pseudomonadota bacterium	1977087	PRJNA946291
SAMN37499272	Yojoa_01_2022_R_epi_K_bin.119	Yojoa_01_2022_R_epi_K_bin.119	Pseudomonadota bacterium	1977087	PRJNA946291
SAMN37499273	Yojoa_01_2022_R_epi_K_bin.121	Yojoa_01_2022_R_epi_K_bin.121	Actinomycetota bacterium	2900548	PRJNA946291
SAMN37499274	Yojoa_01_2022_R_epi_K_bin.21	Yojoa_01_2022_R_epi_K_bin.21	Pseudomonadota bacterium	1977087	PRJNA946291
SAMN37499275	Yojoa_01_2022_R_epi_K_bin.27	Yojoa_01_2022_R_epi_K_bin.27	Bacteroidota bacterium	1898104	PRJNA946291
SAMN37499276	Yojoa_01_2022_R_epi_K_bin.38	Yojoa_01_2022_R_epi_K_bin.38	Planctomycetota bacterium	2026780	PRJNA946291
SAMN37499277	Yojoa_01_2022_R_epi_K_bin.4	Yojoa_01_2022_R_epi_K_bin.4	Pseudomonadota bacterium	1977087	PRJNA946291
SAMN37499278	Yojoa_01_2022_R_epi_K_bin.58	Yojoa_01_2022_R_epi_K_bin.58	Planctomycetota bacterium	2026780	PRJNA946291
SAMN37499279	Yojoa_01_2022_R_epi_K_bin.72	Yojoa_01_2022_R_epi_K_bin.72	Bacteroidota bacterium	1898104	PRJNA946291
SAMN37499280	Yojoa_01_2022_R_epi_K_bin.80	Yojoa_01_2022_R_epi_K_bin.80	Candidatus Parcubacteria bacterium	2762014	PRJNA946291
SAMN37499281	Yojoa_01_2022_R_hypo_A_bin.111	Yojoa_01_2022_R_hypo_A_bin.111	Bacteroidota bacterium	1898104	PRJNA946291
SAMN37499282	Yojoa_01_2022_R_hypo_A_bin.115	Yojoa_01_2022_R_hypo_A_bin.115	Actinomycetota bacterium	2900548	PRJNA946291
SAMN37499283	Yojoa_01_2022_R_hypo_A_bin.136	Yojoa_01_2022_R_hypo_A_bin.136	Bacteroidota bacterium	1898104	PRJNA946291
SAMN37499284	Yojoa_01_2022_R_hypo_A_bin.32	Yojoa_01_2022_R_hypo_A_bin.32	Pseudomonadota bacterium	1977087	PRJNA946291
SAMN37499285	Yojoa_01_2022_R_hypo_A_bin.54	Yojoa_01_2022_R_hypo_A_bin.54	Pseudomonadota bacterium	1977087	PRJNA946291
SAMN37499286	Yojoa_01_2022_R_hypo_A_bin.62	Yojoa_01_2022_R_hypo_A_bin.62	Bacteroidota bacterium	1898104	PRJNA946291
SAMN37499287	Yojoa_01_2022_R_hypo_A_bin.63	Yojoa_01_2022_R_hypo_A_bin.63	Cyanobacteriota bacterium	2847862	PRJNA946291
SAMN37499288	Yojoa_01_2022_R_hypo_A_bin.65	Yojoa_01_2022_R_hypo_A_bin.65	Actinomycetota bacterium	2900548	PRJNA946291
SAMN37499289	Yojoa_01_2022_R_hypo_A_bin.67	Yojoa_01_2022_R_hypo_A_bin.67	Pseudomonadota bacterium	1977087	PRJNA946291
SAMN37499290	Yojoa_01_2022_R_hypo_A_bin.77	Yojoa_01_2022_R_hypo_A_bin.77	Planctomycetota bacterium	2026780	PRJNA946291
SAMN37499291	Yojoa_01_2022_R_hypo_A_bin.81	Yojoa_01_2022_R_hypo_A_bin.81	Pseudomonadota bacterium	1977087	PRJNA946291

SAMN37499292	Yojoa_01_2022_R_hypo_A_bin.99	Yojoa_01_2022_R_hypo_A_bin.99	Pseudomonadota bacterium	1977087	PRJNA946291
SAMN37499293	Yojoa_01_2022_R_hypo_K_bin.105	Yojoa_01_2022_R_hypo_K_bin.105	Verrucomicrobiota bacterium	2026799	PRJNA946291
SAMN37499294	Yojoa_01_2022_R_hypo_K_bin.54	Yojoa_01_2022_R_hypo_K_bin.54	Bacteroidota bacterium	1898104	PRJNA946291
SAMN37499295	Yojoa_01_2022_R_hypo_K_bin.59	Yojoa_01_2022_R_hypo_K_bin.59	Actinomycetota bacterium	2900548	PRJNA946291
SAMN37499296	Yojoa_01_2022_R_hypo_K_bin.60	Yojoa_01_2022_R_hypo_K_bin.60	Pseudomonadota bacterium	1977087	PRJNA946291
SAMN37499297	Yojoa_01_2022_R_hypo_K_bin.72	Yojoa_01_2022_R_hypo_K_bin.72	Bacteroidota bacterium	1898104	PRJNA946291
SAMN37499298	Yojoa_01_2022_R_hypo_K_bin.94	Yojoa_01_2022_R_hypo_K_bin.94	Bacteroidota bacterium	1898104	PRJNA946291
SAMN37499299	Yojoa_06_2019_B_epi_B_bin.101	Yojoa_06_2019_B_epi_B_bin.101	Bacteroidota bacterium	1898104	PRJNA946291
SAMN37499300	Yojoa_06_2019_B_epi_B_bin.113	Yojoa_06_2019_B_epi_B_bin.113	Planctomycetota bacterium	2026780	PRJNA946291
SAMN37499301	Yojoa_06_2019_B_epi_B_bin.117	Yojoa_06_2019_B_epi_B_bin.117	Actinomycetota bacterium	2900548	PRJNA946291
SAMN37499302	Yojoa_06_2019_B_epi_B_bin.121	Yojoa_06_2019_B_epi_B_bin.121	Pseudomonadota bacterium	1977087	PRJNA946291
SAMN37499303	Yojoa_06_2019_B_epi_B_bin.18	Yojoa_06_2019_B_epi_B_bin.18	Pseudomonadota bacterium	1977087	PRJNA946291
SAMN37499304	Yojoa_06_2019_B_epi_B_bin.21	Yojoa_06_2019_B_epi_B_bin.21	Pseudomonadota bacterium	1977087	PRJNA946291
SAMN37499305	Yojoa_06_2019_B_epi_B_bin.26	Yojoa_06_2019_B_epi_B_bin.26	Bacteroidota bacterium	1898104	PRJNA946291
SAMN37499306	Yojoa_06_2019_B_epi_B_bin.28	Yojoa_06_2019_B_epi_B_bin.28	Bacteroidota bacterium	1898104	PRJNA946291
SAMN37499307	Yojoa_06_2019_B_epi_B_bin.31	Yojoa_06_2019_B_epi_B_bin.31	Bacteroidota bacterium	1898104	PRJNA946291
SAMN37499308	Yojoa_06_2019_B_epi_B_bin.34	Yojoa_06_2019_B_epi_B_bin.34	Bacteroidota bacterium	1898104	PRJNA946291
SAMN37499309	Yojoa_06_2019_B_epi_B_bin.35	Yojoa_06_2019_B_epi_B_bin.35	Planctomycetota bacterium	2026780	PRJNA946291
SAMN37499310	Yojoa_06_2019_B_epi_B_bin.4	Yojoa_06_2019_B_epi_B_bin.4	Actinomycetota bacterium	2900548	PRJNA946291
SAMN37499311	Yojoa_06_2019_B_epi_B_bin.56	Yojoa_06_2019_B_epi_B_bin.56	Bacteroidota bacterium	1898104	PRJNA946291
SAMN37499312	Yojoa_06_2019_B_epi_B_bin.57	Yojoa_06_2019_B_epi_B_bin.57	Bacteroidota bacterium	1898104	PRJNA946291
SAMN37499313	Yojoa_06_2019_B_epi_B_bin.65	Yojoa_06_2019_B_epi_B_bin.65	Actinomycetota bacterium	2900548	PRJNA946291
SAMN37499314	Yojoa_06_2019_B_epi_B_bin.68	Yojoa_06_2019_B_epi_B_bin.68	Verrucomicrobiota bacterium	2026799	PRJNA946291
SAMN37499315	Yojoa_06_2019_B_epi_B_bin.7	Yojoa_06_2019_B_epi_B_bin.7	Pseudomonadota bacterium	1977087	PRJNA946291
SAMN37499316	Yojoa_06_2019_B_epi_B_bin.8	Yojoa_06_2019_B_epi_B_bin.8	Pseudomonadota bacterium	1977087	PRJNA946291
SAMN37499317	Yojoa_06_2019_B_epi_B_bin.85	Yojoa_06_2019_B_epi_B_bin.85	Pseudomonadota bacterium	1977087	PRJNA946291
SAMN37499318	Yojoa_06_2019_B_epi_B_bin.94	Yojoa_06_2019_B_epi_B_bin.94	Bacteroidota bacterium	1898104	PRJNA946291
SAMN37499319	Yojoa_06_2019_B_epi_B_bin.95	Yojoa_06_2019_B_epi_B_bin.95	Actinomycetota bacterium	2900548	PRJNA946291
SAMN37499320	Yojoa_06_2019_B_epi_B_bin.96	Yojoa_06_2019_B_epi_B_bin.96	Pseudomonadota bacterium	1977087	PRJNA946291
SAMN37499321	Yojoa_06_2019_B_epi_B_bin.97	Yojoa_06_2019_B_epi_B_bin.97	Pseudomonadota bacterium	1977087	PRJNA946291
SAMN37499322	Yojoa_06_2019_B_epi_B_bin.98	Yojoa_06_2019_B_epi_B_bin.98	Pseudomonadota bacterium	1977087	PRJNA946291
SAMN37499323	Yojoa_06_2019_B_hypo_B_bin.1	Yojoa_06_2019_B_hypo_B_bin.1	Actinomycetota bacterium	2900548	PRJNA946291
SAMN37499324	Yojoa_06_2019_B_hypo_B_bin.106	Yojoa_06_2019_B_hypo_B_bin.106	Bacteroidota bacterium	1898104	PRJNA946291
SAMN37499325	Yojoa_06_2019_B_hypo_B_bin.107	Yojoa_06_2019_B_hypo_B_bin.107	Verrucomicrobiota bacterium	2026799	PRJNA946291
SAMN37499326	Yojoa_06_2019_B_hypo_B_bin.108	Yojoa_06_2019_B_hypo_B_bin.108	Verrucomicrobiota bacterium	2026799	PRJNA946291
SAMN37499327	Yojoa_06_2019_B_hypo_B_bin.11	Yojoa_06_2019_B_hypo_B_bin.11	Candidatus Omnitrophota bacterium	2035772	PRJNA946291

SAMN37499328	Yojoa_06_2019_B_hypo_B_bin.110	Yojoa_06_2019_B_hypo_B_bin.110	Candidatus Parcubacteria bacterium	2762014	PRJNA946291
SAMN37499329	Yojoa_06_2019_B_hypo_B_bin.111	Yojoa_06_2019_B_hypo_B_bin.111	Pseudomonadota bacterium	1977087	PRJNA946291
SAMN37499330	Yojoa_06_2019_B_hypo_B_bin.114	Yojoa_06_2019_B_hypo_B_bin.114	Bacillota bacterium	1879010	PRJNA946291
SAMN37499331	Yojoa_06_2019_B_hypo_B_bin.115	Yojoa_06_2019_B_hypo_B_bin.115	Candidatus Latescibacteria bacterium	2053570	PRJNA946291
SAMN37499332	Yojoa_06_2019_B_hypo_B_bin.121	Yojoa_06_2019_B_hypo_B_bin.121	Candidatus Parcubacteria bacterium	2762014	PRJNA946291
SAMN37499333	Yojoa_06_2019_B_hypo_B_bin.13	Yojoa_06_2019_B_hypo_B_bin.13	Candidatus Omnitrophota bacterium	2035772	PRJNA946291
SAMN37499334	Yojoa_06_2019_B_hypo_B_bin.130	Yojoa_06_2019_B_hypo_B_bin.130	Candidatus Omnitrophota bacterium	2035772	PRJNA946291
SAMN37499335	Yojoa_06_2019_B_hypo_B_bin.131	Yojoa_06_2019_B_hypo_B_bin.131	Verrucomicrobiota bacterium	2026799	PRJNA946291
SAMN37499336	Yojoa_06_2019_B_hypo_B_bin.14	Yojoa_06_2019_B_hypo_B_bin.14	Candidatus Parcubacteria bacterium	2762014	PRJNA946291
SAMN37499337	Yojoa_06_2019_B_hypo_B_bin.146	Yojoa_06_2019_B_hypo_B_bin.146	Planctomycetota bacterium	2026780	PRJNA946291
SAMN37499338	Yojoa_06_2019_B_hypo_B_bin.156	Yojoa_06_2019_B_hypo_B_bin.156	Actinomycetota bacterium	2900548	PRJNA946291
SAMN37499339	Yojoa_06_2019_B_hypo_B_bin.158	Yojoa_06_2019_B_hypo_B_bin.158	Pseudomonadota bacterium	1977087	PRJNA946291
SAMN37499340	Yojoa_06_2019_B_hypo_B_bin.160	Yojoa_06_2019_B_hypo_B_bin.160	Candidatus Bathyarchaeota archaeon	2026714	PRJNA946291
SAMN37499341	Yojoa_06_2019_B_hypo_B_bin.161	Yojoa_06_2019_B_hypo_B_bin.161	Nanoarchaeota archaeon	2026764	PRJNA946291
SAMN37499342	Yojoa_06_2019_B_hypo_B_bin.162	Yojoa_06_2019_B_hypo_B_bin.162	Chloroflexota bacterium	2026724	PRJNA946291
SAMN37499343	Yojoa_06_2019_B_hypo_B_bin.17	Yojoa_06_2019_B_hypo_B_bin.17	Candidatus Woesearchaeota archaeon	2026803	PRJNA946291
SAMN37499344	Yojoa_06_2019_B_hypo_B_bin.171	Yojoa_06_2019_B_hypo_B_bin.171	Pseudomonadota bacterium	1977087	PRJNA946291
SAMN37499345	Yojoa_06_2019_B_hypo_B_bin.172	Yojoa_06_2019_B_hypo_B_bin.172	Bacteroidota bacterium	1898104	PRJNA946291
SAMN37499346	Yojoa_06_2019_B_hypo_B_bin.175	Yojoa_06_2019_B_hypo_B_bin.175	Candidatus Cloacimonadota bacterium	2030808	PRJNA946291
SAMN37499347	Yojoa_06_2019_B_hypo_B_bin.18	Yojoa_06_2019_B_hypo_B_bin.18	Chloroflexota bacterium	2026724	PRJNA946291
SAMN37499348	Yojoa_06_2019_B_hypo_B_bin.181	Yojoa_06_2019_B_hypo_B_bin.181	Chloroflexota bacterium	2026724	PRJNA946291
SAMN37499349	Yojoa_06_2019_B_hypo_B_bin.183	Yojoa_06_2019_B_hypo_B_bin.183	Chloroflexota bacterium	2026724	PRJNA946291
SAMN37499350	Yojoa_06_2019_B_hypo_B_bin.2	Yojoa_06_2019_B_hypo_B_bin.2	Bacteroidota bacterium	1898104	PRJNA946291
SAMN37499351	Yojoa_06_2019_B_hypo_B_bin.22	Yojoa_06_2019_B_hypo_B_bin.22	Chloroflexota bacterium	2026724	PRJNA946291
SAMN37499352	Yojoa_06_2019_B_hypo_B_bin.24	Yojoa_06_2019_B_hypo_B_bin.24	Verrucomicrobiota bacterium	2026799	PRJNA946291
SAMN37499353	Yojoa_06_2019_B_hypo_B_bin.25	Yojoa_06_2019_B_hypo_B_bin.25	Nanoarchaeota archaeon	2026764	PRJNA946291
SAMN37499354	Yojoa_06_2019_B_hypo_B_bin.32	Yojoa_06_2019_B_hypo_B_bin.32	Pseudomonadota bacterium	1977087	PRJNA946291
SAMN37499355	Yojoa_06_2019_B_hypo_B_bin.40	Yojoa_06_2019_B_hypo_B_bin.40	candidate division NC10 bacterium	2072417	PRJNA946291
SAMN37499356	Yojoa_06_2019_B_hypo_B_bin.47	Yojoa_06_2019_B_hypo_B_bin.47	Nanoarchaeota archaeon	2026764	PRJNA946291
SAMN37499357	Yojoa_06_2019_B_hypo_B_bin.50	Yojoa_06_2019_B_hypo_B_bin.50	Actinomycetota bacterium	2900548	PRJNA946291
SAMN37499358	Yojoa_06_2019_B_hypo_B_bin.51	Yojoa_06_2019_B_hypo_B_bin.51	Verrucomicrobiota bacterium	2026799	PRJNA946291
SAMN37499359	Yojoa_06_2019_B_hypo_B_bin.55	Yojoa_06_2019_B_hypo_B_bin.55	Candidatus Parcubacteria bacterium	2762014	PRJNA946291
SAMN37499360	Yojoa_06_2019_B_hypo_B_bin.57	Yojoa_06_2019_B_hypo_B_bin.57	Bacteroidota bacterium	1898104	PRJNA946291
SAMN37499361	Yojoa_06_2019_B_hypo_B_bin.60	Yojoa_06_2019_B_hypo_B_bin.60	Nanoarchaeota archaeon	2026764	PRJNA946291
SAMN37499362	Yojoa_06_2019_B_hypo_B_bin.62	Yojoa_06_2019_B_hypo_B_bin.62	Pseudomonadota bacterium	1977087	PRJNA946291
SAMN37499363	Yojoa_06_2019_B_hypo_B_bin.66	Yojoa_06_2019_B_hypo_B_bin.66	bacterium	1869227	PRJNA946291

SAMN37499364	Yojoa_06_2019_B_hypo_B_bin.67	Yojoa_06_2019_B_hypo_B_bin.67	Pseudomonadota bacterium	1977087	PRJNA946291
SAMN37499365	Yojoa_06_2019_B_hypo_B_bin.7	Yojoa_06_2019_B_hypo_B_bin.7	Bacteroidota bacterium	1898104	PRJNA946291
SAMN37499366	Yojoa_06_2019_B_hypo_B_bin.70	Yojoa_06_2019_B_hypo_B_bin.70	Bacteroidota bacterium	1898104	PRJNA946291
SAMN37499367	Yojoa_06_2019_B_hypo_B_bin.73	Yojoa_06_2019_B_hypo_B_bin.73	Chloroflexota bacterium	2026724	PRJNA946291
SAMN37499368	Yojoa_06_2019_B_hypo_B_bin.74	Yojoa_06_2019_B_hypo_B_bin.74	Planctomycetota bacterium	2026780	PRJNA946291
SAMN37499369	Yojoa_06_2019_B_hypo_B_bin.78	Yojoa_06_2019_B_hypo_B_bin.78	Pseudomonadota bacterium	1977087	PRJNA946291
SAMN37499370	Yojoa_06_2019_B_hypo_B_bin.81	Yojoa_06_2019_B_hypo_B_bin.81	Pseudomonadota bacterium	1977087	PRJNA946291
SAMN37499371	Yojoa_06_2019_B_hypo_B_bin.82	Yojoa_06_2019_B_hypo_B_bin.82	Planctomycetota bacterium	2026780	PRJNA946291
SAMN37499372	Yojoa_06_2019_B_hypo_B_bin.85	Yojoa_06_2019_B_hypo_B_bin.85	Verrucomicrobiota bacterium	2026799	PRJNA946291
SAMN37499373	Yojoa_06_2019_B_hypo_B_bin.87	Yojoa_06_2019_B_hypo_B_bin.87	Bacteroidota bacterium	1898104	PRJNA946291
SAMN37499374	Yojoa_06_2019_B_hypo_B_bin.89	Yojoa_06_2019_B_hypo_B_bin.89	Pseudomonadota bacterium	1977087	PRJNA946291
SAMN37499375	Yojoa_06_2019_B_hypo_B_bin.9	Yojoa_06_2019_B_hypo_B_bin.9	Bacteroidota bacterium	1898104	PRJNA946291
SAMN37499376	Yojoa_06_2019_B_hypo_B_bin.90	Yojoa_06_2019_B_hypo_B_bin.90	Pseudomonadota bacterium	1977087	PRJNA946291
SAMN37499377	Yojoa_06_2019_B_hypo_B_bin.91	Yojoa_06_2019_B_hypo_B_bin.91	Bacteroidota bacterium	1898104	PRJNA946291
SAMN37499378	Yojoa_06_2019_B_hypo_B_bin.92	Yojoa_06_2019_B_hypo_B_bin.92	Candidatus Parcubacteria bacterium	2762014	PRJNA946291
SAMN37499379	Yojoa_06_2021_B_epi_A_bin.102	Yojoa_06_2021_B_epi_A_bin.102	Pseudomonadota bacterium	1977087	PRJNA946291
SAMN37499380	Yojoa_06_2021_B_epi_A_bin.109	Yojoa_06_2021_B_epi_A_bin.109	Planctomycetota bacterium	2026780	PRJNA946291
SAMN37499381	Yojoa_06_2021_B_epi_A_bin.122	Yojoa_06_2021_B_epi_A_bin.122	Bacteroidota bacterium	1898104	PRJNA946291
SAMN37499382	Yojoa_06_2021_B_epi_A_bin.135	Yojoa_06_2021_B_epi_A_bin.135	Pseudomonadota bacterium	1977087	PRJNA946291
SAMN37499383	Yojoa_06_2021_B_epi_A_bin.137	Yojoa_06_2021_B_epi_A_bin.137	Bacteroidota bacterium	1898104	PRJNA946291
SAMN37499384	Yojoa_06_2021_B_epi_A_bin.14	Yojoa_06_2021_B_epi_A_bin.14	Bacteroidota bacterium	1898104	PRJNA946291
SAMN37499385	Yojoa_06_2021_B_epi_A_bin.142	Yojoa_06_2021_B_epi_A_bin.142	Verrucomicrobiota bacterium	2026799	PRJNA946291
SAMN37499386	Yojoa_06_2021_B_epi_A_bin.18	Yojoa_06_2021_B_epi_A_bin.18	Pseudomonadota bacterium	1977087	PRJNA946291
SAMN37499387	Yojoa_06_2021_B_epi_A_bin.32	Yojoa_06_2021_B_epi_A_bin.32	Verrucomicrobiota bacterium	2026799	PRJNA946291
SAMN37499388	Yojoa_06_2021_B_epi_A_bin.40	Yojoa_06_2021_B_epi_A_bin.40	Verrucomicrobiota bacterium	2026799	PRJNA946291
SAMN37499389	Yojoa_06_2021_B_epi_A_bin.44	Yojoa_06_2021_B_epi_A_bin.44	Pseudomonadota bacterium	1977087	PRJNA946291
SAMN37499390	Yojoa_06_2021_B_epi_A_bin.48	Yojoa_06_2021_B_epi_A_bin.48	Actinomycetota bacterium	2900548	PRJNA946291
SAMN37499391	Yojoa_06_2021_B_epi_A_bin.49	Yojoa_06_2021_B_epi_A_bin.49	Pseudomonadota bacterium	1977087	PRJNA946291
SAMN37499392	Yojoa_06_2021_B_epi_A_bin.50	Yojoa_06_2021_B_epi_A_bin.50	Verrucomicrobiota bacterium	2026799	PRJNA946291
SAMN37499393	Yojoa_06_2021_B_epi_A_bin.59	Yojoa_06_2021_B_epi_A_bin.59	Pseudomonadota bacterium	1977087	PRJNA946291
SAMN37499394	Yojoa_06_2021_B_epi_A_bin.64	Yojoa_06_2021_B_epi_A_bin.64	Bacteroidota bacterium	1898104	PRJNA946291
SAMN37499395	Yojoa_06_2021_B_epi_A_bin.70	Yojoa_06_2021_B_epi_A_bin.70	Planctomycetota bacterium	2026780	PRJNA946291
SAMN37499396	Yojoa_06_2021_B_epi_A_bin.72	Yojoa_06_2021_B_epi_A_bin.72	Pseudomonadota bacterium	1977087	PRJNA946291
SAMN37499397	Yojoa_06_2021_B_epi_A_bin.76	Yojoa_06_2021_B_epi_A_bin.76	Verrucomicrobiota bacterium	2026799	PRJNA946291
SAMN37499398	Yojoa_06_2021_B_epi_A_bin.81	Yojoa_06_2021_B_epi_A_bin.81	Planctomycetota bacterium	2026780	PRJNA946291
SAMN37499399	Yojoa_06_2021_B_epi_K_bin.105	Yojoa_06_2021_B_epi_K_bin.105	Bacteroidota bacterium	1898104	PRJNA946291

SAMN37499400	Yojoa_06_2021_B_epi_K_bin.112	Yojoa_06_2021_B_epi_K_bin.112	Bacteroidota bacterium	1898104	PRJNA946291
SAMN37499401	Yojoa_06_2021_B_epi_K_bin.40	Yojoa_06_2021_B_epi_K_bin.40	Pseudomonadota bacterium	1977087	PRJNA946291
SAMN37499402	Yojoa_06_2021_B_epi_K_bin.41	Yojoa_06_2021_B_epi_K_bin.41	Bacteroidota bacterium	1898104	PRJNA946291
SAMN37499403	Yojoa_06_2021_B_epi_K_bin.45	Yojoa_06_2021_B_epi_K_bin.45	Chloroflexota bacterium	2026724	PRJNA946291
SAMN37499404	Yojoa_06_2021_B_epi_K_bin.51	Yojoa_06_2021_B_epi_K_bin.51	Bacteroidota bacterium	1898104	PRJNA946291
SAMN37499405	Yojoa_06_2021_B_epi_K_bin.85	Yojoa_06_2021_B_epi_K_bin.85	Pseudomonadota bacterium	1977087	PRJNA946291
SAMN37499406	Yojoa_06_2021_B_epi_K_bin.86	Yojoa_06_2021_B_epi_K_bin.86	Bacteroidota bacterium	1898104	PRJNA946291
SAMN37499407	Yojoa_06_2021_B_epi_K_bin.99	Yojoa_06_2021_B_epi_K_bin.99	Planctomycetota bacterium	2026780	PRJNA946291
SAMN37499408	Yojoa_06_2021_B_hypo_A_bin.107	Yojoa_06_2021_B_hypo_A_bin.107	Planctomycetota bacterium	2026780	PRJNA946291
SAMN37499409	Yojoa_06_2021_B_hypo_A_bin.112	Yojoa_06_2021_B_hypo_A_bin.112	Pseudomonadota bacterium	1977087	PRJNA946291
SAMN37499410	Yojoa_06_2021_B_hypo_A_bin.127	Yojoa_06_2021_B_hypo_A_bin.127	Cyanobacteriota bacterium	2847862	PRJNA946291
SAMN37499411	Yojoa_06_2021_B_hypo_A_bin.128	Yojoa_06_2021_B_hypo_A_bin.128	Planctomycetota bacterium	2026780	PRJNA946291
SAMN37499412	Yojoa_06_2021_B_hypo_A_bin.130	Yojoa_06_2021_B_hypo_A_bin.130	Fibrobacterota bacterium	2052160	PRJNA946291
SAMN37499413	Yojoa_06_2021_B_hypo_A_bin.135	Yojoa_06_2021_B_hypo_A_bin.135	Pseudomonadota bacterium	1977087	PRJNA946291
SAMN37499414	Yojoa_06_2021_B_hypo_A_bin.14	Yojoa_06_2021_B_hypo_A_bin.14	Candidatus Hydrogenedentes bacterium	2030809	PRJNA946291
SAMN37499415	Yojoa_06_2021_B_hypo_A_bin.142	Yojoa_06_2021_B_hypo_A_bin.142	Actinomycetota bacterium	2900548	PRJNA946291
SAMN37499416	Yojoa_06_2021_B_hypo_A_bin.143	Yojoa_06_2021_B_hypo_A_bin.143	Candidatus Parcubacteria bacterium	2762014	PRJNA946291
SAMN37499417	Yojoa_06_2021_B_hypo_A_bin.148	Yojoa_06_2021_B_hypo_A_bin.148	Pseudomonadota bacterium	1977087	PRJNA946291
SAMN37499418	Yojoa_06_2021_B_hypo_A_bin.149	Yojoa_06_2021_B_hypo_A_bin.149	Planctomycetota bacterium	2026780	PRJNA946291
SAMN37499419	Yojoa_06_2021_B_hypo_A_bin.150	Yojoa_06_2021_B_hypo_A_bin.150	Planctomycetota bacterium	2026780	PRJNA946291
SAMN37499420	Yojoa_06_2021_B_hypo_A_bin.158	Yojoa_06_2021_B_hypo_A_bin.158	Bacillota bacterium	1879010	PRJNA946291
SAMN37499421	Yojoa_06_2021_B_hypo_A_bin.2	Yojoa_06_2021_B_hypo_A_bin.2	Pseudomonadota bacterium	1977087	PRJNA946291
SAMN37499422	Yojoa_06_2021_B_hypo_A_bin.24	Yojoa_06_2021_B_hypo_A_bin.24	Pseudomonadota bacterium	1977087	PRJNA946291
SAMN37499423	Yojoa_06_2021_B_hypo_A_bin.26	Yojoa_06_2021_B_hypo_A_bin.26	Planctomycetota bacterium	2026780	PRJNA946291
SAMN37499424	Yojoa_06_2021_B_hypo_A_bin.33	Yojoa_06_2021_B_hypo_A_bin.33	Bacillota bacterium	1879010	PRJNA946291
SAMN37499425	Yojoa_06_2021_B_hypo_A_bin.40	Yojoa_06_2021_B_hypo_A_bin.40	Verrucomicrobiota bacterium	2026799	PRJNA946291
SAMN37499426	Yojoa_06_2021_B_hypo_A_bin.43	Yojoa_06_2021_B_hypo_A_bin.43	Actinomycetota bacterium	2900548	PRJNA946291
SAMN37499427	Yojoa_06_2021_B_hypo_A_bin.57	Yojoa_06_2021_B_hypo_A_bin.57	Planctomycetota bacterium	2026780	PRJNA946291
SAMN37499428	Yojoa_06_2021_B_hypo_A_bin.58	Yojoa_06_2021_B_hypo_A_bin.58	Verrucomicrobiota bacterium	2026799	PRJNA946291
SAMN37499429	Yojoa_06_2021_B_hypo_A_bin.61	Yojoa_06_2021_B_hypo_A_bin.61	Verrucomicrobiota bacterium	2026799	PRJNA946291
SAMN37499430	Yojoa_06_2021_B_hypo_A_bin.64	Yojoa_06_2021_B_hypo_A_bin.64	Pseudomonadota bacterium	1977087	PRJNA946291
SAMN37499431	Yojoa_06_2021_B_hypo_A_bin.66	Yojoa_06_2021_B_hypo_A_bin.66	Pseudomonadota bacterium	1977087	PRJNA946291
SAMN37499432	Yojoa_06_2021_B_hypo_A_bin.67	Yojoa_06_2021_B_hypo_A_bin.67	Pseudomonadota bacterium	1977087	PRJNA946291
SAMN37499433	Yojoa_06_2021_B_hypo_A_bin.71	Yojoa_06_2021_B_hypo_A_bin.71	Cyanobacteriota bacterium	2847862	PRJNA946291
SAMN37499434	Yojoa_06_2021_B_hypo_A_bin.75	Yojoa_06_2021_B_hypo_A_bin.75	Chloroflexota bacterium	2026724	PRJNA946291
SAMN37499435	Yojoa_06_2021_B_hypo_A_bin.77	Yojoa_06_2021_B_hypo_A_bin.77	Pseudomonadota bacterium	1977087	PRJNA946291

SAMN37499436	Yojoa_06_2021_B_hypo_A_bin.80	Yojoa_06_2021_B_hypo_A_bin.80	Caldisericota bacterium	2052147	PRJNA946291
SAMN37499437	Yojoa_06_2021_B_hypo_A_bin.87	Yojoa_06_2021_B_hypo_A_bin.87	Bacillota bacterium	1879010	PRJNA946291
SAMN37499438	Yojoa_06_2021_B_hypo_A_bin.88	Yojoa_06_2021_B_hypo_A_bin.88	Cyanobacteriota bacterium	2847862	PRJNA946291
SAMN37499439	Yojoa_06_2021_B_hypo_A_bin.89	Yojoa_06_2021_B_hypo_A_bin.89	Candidatus Parcubacteria bacterium	2762014	PRJNA946291
SAMN37499440	Yojoa_06_2021_B_hypo_A_bin.9	Yojoa_06_2021_B_hypo_A_bin.9	Planctomycetota bacterium	2026780	PRJNA946291
SAMN37499441	Yojoa_06_2021_B_hypo_A_bin.91	Yojoa_06_2021_B_hypo_A_bin.91	Gemmatimonadota bacterium	2026742	PRJNA946291
SAMN37499442	Yojoa_06_2021_B_hypo_A_bin.93	Yojoa_06_2021_B_hypo_A_bin.93	Candidatus Parcubacteria bacterium	2762014	PRJNA946291
SAMN37499443	Yojoa_06_2021_B_hypo_A_bin.99	Yojoa_06_2021_B_hypo_A_bin.99	Candidatus Thermoplasmatota archaeon	2806419	PRJNA946291
SAMN37499444	Yojoa_06_2021_B_hypo_K_bin.106	Yojoa_06_2021_B_hypo_K_bin.106	Acidobacteriota bacterium	1978231	PRJNA946291
SAMN37499445	Yojoa_06_2021_B_hypo_K_bin.14	Yojoa_06_2021_B_hypo_K_bin.14	Chloroflexota bacterium	2026724	PRJNA946291
SAMN37499446	Yojoa_06_2021_B_hypo_K_bin.15	Yojoa_06_2021_B_hypo_K_bin.15	Pseudomonadota bacterium	1977087	PRJNA946291
SAMN37499447	Yojoa_06_2021_B_hypo_K_bin.27	Yojoa_06_2021_B_hypo_K_bin.27	Chloroflexota bacterium	2026724	PRJNA946291
SAMN37499448	Yojoa_06_2021_B_hypo_K_bin.36	Yojoa_06_2021_B_hypo_K_bin.36	Bacteroidota bacterium	1898104	PRJNA946291
SAMN37499449	Yojoa_06_2021_B_hypo_K_bin.46	Yojoa_06_2021_B_hypo_K_bin.46	Pseudomonadota bacterium	1977087	PRJNA946291
SAMN37499450	Yojoa_06_2021_B_hypo_K_bin.51	Yojoa_06_2021_B_hypo_K_bin.51	Candidatus Sumerlaeota bacterium	2107588	PRJNA946291
SAMN37499451	Yojoa_06_2021_B_hypo_K_bin.52	Yojoa_06_2021_B_hypo_K_bin.52	Verrucomicrobiota bacterium	2026799	PRJNA946291
SAMN37499452	Yojoa_06_2021_B_hypo_K_bin.53	Yojoa_06_2021_B_hypo_K_bin.53	Chloroflexota bacterium	2026724	PRJNA946291
SAMN37499453	Yojoa_06_2021_B_hypo_K_bin.57	Yojoa_06_2021_B_hypo_K_bin.57	Bacillota bacterium	1879010	PRJNA946291
SAMN37499454	Yojoa_06_2021_B_hypo_K_bin.63	Yojoa_06_2021_B_hypo_K_bin.63	Cyanobacteriota bacterium	2847862	PRJNA946291
SAMN37499455	Yojoa_06_2021_B_hypo_K_bin.66	Yojoa_06_2021_B_hypo_K_bin.66	Pseudomonadota bacterium	1977087	PRJNA946291
SAMN37499456	Yojoa_06_2021_B_hypo_K_bin.74	Yojoa_06_2021_B_hypo_K_bin.74	Bacteroidota bacterium	1898104	PRJNA946291
SAMN37499457	Yojoa_06_2021_B_hypo_K_bin.78	Yojoa_06_2021_B_hypo_K_bin.78	Pseudomonadota bacterium	1977087	PRJNA946291
SAMN37499458	Yojoa_06_2021_B_hypo_K_bin.91	Yojoa_06_2021_B_hypo_K_bin.91	Bacillota bacterium	1879010	PRJNA946291
SAMN37499459	Yojoa_06_2021_B_hypo_K_bin.96	Yojoa_06_2021_B_hypo_K_bin.96	Chloroflexota bacterium	2026724	PRJNA946291
SAMN37499460	Yojoa_06_2021_E_epi_A_bin.102	Yojoa_06_2021_E_epi_A_bin.102	Planctomycetota bacterium	2026780	PRJNA946291
SAMN37499461	Yojoa_06_2021_E_epi_A_bin.106	Yojoa_06_2021_E_epi_A_bin.106	Planctomycetota bacterium	2026780	PRJNA946291
SAMN37499462	Yojoa_06_2021_E_epi_A_bin.112	Yojoa_06_2021_E_epi_A_bin.112	Pseudomonadota bacterium	1977087	PRJNA946291
SAMN37499463	Yojoa_06_2021_E_epi_A_bin.120	Yojoa_06_2021_E_epi_A_bin.120	Bacteroidota bacterium	1898104	PRJNA946291
SAMN37499464	Yojoa_06_2021_E_epi_A_bin.139	Yojoa_06_2021_E_epi_A_bin.139	Pseudomonadota bacterium	1977087	PRJNA946291
SAMN37499465	Yojoa_06_2021_E_epi_A_bin.140	Yojoa_06_2021_E_epi_A_bin.140	Bacteroidota bacterium	1898104	PRJNA946291
SAMN37499466	Yojoa_06_2021_E_epi_A_bin.143	Yojoa_06_2021_E_epi_A_bin.143	Verrucomicrobiota bacterium	2026799	PRJNA946291
SAMN37499467	Yojoa_06_2021_E_epi_A_bin.148	Yojoa_06_2021_E_epi_A_bin.148	Pseudomonadota bacterium	1977087	PRJNA946291
SAMN37499468	Yojoa_06_2021_E_epi_A_bin.149	Yojoa_06_2021_E_epi_A_bin.149	Pseudomonadota bacterium	1977087	PRJNA946291
SAMN37499469	Yojoa_06_2021_E_epi_A_bin.19	Yojoa_06_2021_E_epi_A_bin.19	Bacteroidota bacterium	1898104	PRJNA946291
SAMN37499470	Yojoa_06_2021_E_epi_A_bin.42	Yojoa_06_2021_E_epi_A_bin.42	Chloroflexota bacterium	2026724	PRJNA946291
SAMN37499471	Yojoa_06_2021_E_epi_A_bin.43	Yojoa_06_2021_E_epi_A_bin.43	Planctomycetota bacterium	2026780	PRJNA946291

SAMN37499472	Yojoa_06_2021_E_epi_A_bin.47	Yojoa_06_2021_E_epi_A_bin.47	Cyanobacteriota bacterium	2847862	PRJNA946291
SAMN37499473	Yojoa_06_2021_E_epi_A_bin.48	Yojoa_06_2021_E_epi_A_bin.48	Bacteroidota bacterium	1898104	PRJNA946291
SAMN37499474	Yojoa_06_2021_E_epi_A_bin.62	Yojoa_06_2021_E_epi_A_bin.62	Bacteroidota bacterium	1898104	PRJNA946291
SAMN37499475	Yojoa_06_2021_E_epi_A_bin.70	Yojoa_06_2021_E_epi_A_bin.70	Pseudomonadota bacterium	1977087	PRJNA946291
SAMN37499476	Yojoa_06_2021_E_epi_A_bin.88	Yojoa_06_2021_E_epi_A_bin.88	Pseudomonadota bacterium	1977087	PRJNA946291
SAMN37499477	Yojoa_06_2021_E_epi_A_bin.94	Yojoa_06_2021_E_epi_A_bin.94	Pseudomonadota bacterium	1977087	PRJNA946291
SAMN37499478	Yojoa_06_2021_E_epi_A_bin.98	Yojoa_06_2021_E_epi_A_bin.98	Planctomycetota bacterium	2026780	PRJNA946291
SAMN37499479	Yojoa_06_2021_E_epi_K_bin.102	Yojoa_06_2021_E_epi_K_bin.102	Planctomycetota bacterium	2026780	PRJNA946291
SAMN37499480	Yojoa_06_2021_E_epi_K_bin.15	Yojoa_06_2021_E_epi_K_bin.15	Pseudomonadota bacterium	1977087	PRJNA946291
SAMN37499481	Yojoa_06_2021_E_epi_K_bin.19	Yojoa_06_2021_E_epi_K_bin.19	Verrucomicrobiota bacterium	2026799	PRJNA946291
SAMN37499482	Yojoa_06_2021_E_epi_K_bin.25	Yojoa_06_2021_E_epi_K_bin.25	Actinomycetota bacterium	2900548	PRJNA946291
SAMN37499483	Yojoa_06_2021_E_epi_K_bin.35	Yojoa_06_2021_E_epi_K_bin.35	Bacteroidota bacterium	1898104	PRJNA946291
SAMN37499484	Yojoa_06_2021_E_epi_K_bin.37	Yojoa_06_2021_E_epi_K_bin.37	Bacteroidota bacterium	1898104	PRJNA946291
SAMN37499485	Yojoa_06_2021_E_epi_K_bin.46	Yojoa_06_2021_E_epi_K_bin.46	Bacteroidota bacterium	1898104	PRJNA946291
SAMN37499486	Yojoa_06_2021_E_epi_K_bin.49	Yojoa_06_2021_E_epi_K_bin.49	Acidobacteriota bacterium	1978231	PRJNA946291
SAMN37499487	Yojoa_06_2021_E_epi_K_bin.6	Yojoa_06_2021_E_epi_K_bin.6	Actinomycetota bacterium	2900548	PRJNA946291
SAMN37499488	Yojoa_06_2021_E_epi_K_bin.62	Yojoa_06_2021_E_epi_K_bin.62	Planctomycetota bacterium	2026780	PRJNA946291
SAMN37499489	Yojoa_06_2021_E_epi_K_bin.75	Yojoa_06_2021_E_epi_K_bin.75	Pseudomonadota bacterium	1977087	PRJNA946291
SAMN37499490	Yojoa_06_2021_E_epi_K_bin.8	Yojoa_06_2021_E_epi_K_bin.8	Acidobacteriota bacterium	1978231	PRJNA946291
SAMN37499491	Yojoa_06_2021_E_epi_K_bin.83	Yojoa_06_2021_E_epi_K_bin.83	Pseudomonadota bacterium	1977087	PRJNA946291
SAMN37499492	Yojoa_06_2021_E_epi_K_bin.85	Yojoa_06_2021_E_epi_K_bin.85	Bacteroidota bacterium	1898104	PRJNA946291
SAMN37499493	Yojoa_06_2021_E_hypo_A_bin.2	Yojoa_06_2021_E_hypo_A_bin.2	Verrucomicrobiota bacterium	2026799	PRJNA946291
SAMN37499494	Yojoa_06_2021_E_hypo_A_bin.22	Yojoa_06_2021_E_hypo_A_bin.22	Candidatus Delongbacteria bacterium	2044594	PRJNA946291
SAMN37499495	Yojoa_06_2021_E_hypo_A_bin.31	Yojoa_06_2021_E_hypo_A_bin.31	Planctomycetota bacterium	2026780	PRJNA946291
SAMN37499496	Yojoa_06_2021_E_hypo_A_bin.33	Yojoa_06_2021_E_hypo_A_bin.33	Pseudomonadota bacterium	1977087	PRJNA946291
SAMN37499497	Yojoa_06_2021_E_hypo_A_bin.65	Yojoa_06_2021_E_hypo_A_bin.65	Verrucomicrobiota bacterium	2026799	PRJNA946291
SAMN37499498	Yojoa_06_2021_E_hypo_A_bin.66	Yojoa_06_2021_E_hypo_A_bin.66	Candidatus Delongbacteria bacterium	2044594	PRJNA946291
SAMN37499499	Yojoa_06_2021_E_hypo_A_bin.72	Yojoa_06_2021_E_hypo_A_bin.72	Verrucomicrobiota bacterium	2026799	PRJNA946291
SAMN37499500	Yojoa_06_2021_E_hypo_A_bin.8	Yojoa_06_2021_E_hypo_A_bin.8	Chloroflexota bacterium	2026724	PRJNA946291
SAMN37499501	Yojoa_06_2021_E_hypo_A_bin.82	Yojoa_06_2021_E_hypo_A_bin.82	Chloroflexota bacterium	2026724	PRJNA946291
SAMN37499502	Yojoa_06_2021_E_hypo_A_bin.86	Yojoa_06_2021_E_hypo_A_bin.86	Caldisericota bacterium	2052147	PRJNA946291
SAMN37499503	Yojoa_06_2021_E_hypo_A_bin.97	Yojoa_06_2021_E_hypo_A_bin.97	Bacteroidota bacterium	1898104	PRJNA946291
SAMN37499504	Yojoa_06_2021_E_hypo_K_bin.26	Yojoa_06_2021_E_hypo_K_bin.26	Chloroflexota bacterium	2026724	PRJNA946291
SAMN37499505	Yojoa_06_2021_E_hypo_K_bin.33	Yojoa_06_2021_E_hypo_K_bin.33	Verrucomicrobiota bacterium	2026799	PRJNA946291
SAMN37499506	Yojoa_06_2021_E_hypo_K_bin.39	Yojoa_06_2021_E_hypo_K_bin.39	Verrucomicrobiota bacterium	2026799	PRJNA946291
SAMN37499507	Yojoa_06_2021_E_hypo_K_bin.43	Yojoa_06_2021_E_hypo_K_bin.43	Chloroflexota bacterium	2026724	PRJNA946291

SAMN37499508	Yojoa_06_2021_E_hypo_K_bin.53	Yojoa_06_2021_E_hypo_K_bin.53	Actinomycetota bacterium	2900548	PRJNA946291
SAMN37499509	Yojoa_06_2021_E_hypo_K_bin.69	Yojoa_06_2021_E_hypo_K_bin.69	Verrucomicrobiota bacterium	2026799	PRJNA946291
SAMN37499510	Yojoa_06_2021_E_hypo_K_bin.73	Yojoa_06_2021_E_hypo_K_bin.73	Chloroflexota bacterium	2026724	PRJNA946291
SAMN37499511	Yojoa_06_2021_E_hypo_K_bin.79	Yojoa_06_2021_E_hypo_K_bin.79	Chloroflexota bacterium	2026724	PRJNA946291
SAMN37499512	Yojoa_06_2021_E_hypo_K_bin.81	Yojoa_06_2021_E_hypo_K_bin.81	Pseudomonadota bacterium	1977087	PRJNA946291
SAMN37499513	Yojoa_06_2021_R_epi_A_bin.119	Yojoa_06_2021_R_epi_A_bin.119	Actinomycetota bacterium	2900548	PRJNA946291
SAMN37499514	Yojoa_06_2021_R_epi_A_bin.125	Yojoa_06_2021_R_epi_A_bin.125	Pseudomonadota bacterium	1977087	PRJNA946291
SAMN37499515	Yojoa_06_2021_R_epi_A_bin.14	Yojoa_06_2021_R_epi_A_bin.14	Acidobacteriota bacterium	1978231	PRJNA946291
SAMN37499516	Yojoa_06_2021_R_epi_A_bin.51	Yojoa_06_2021_R_epi_A_bin.51	Pseudomonadota bacterium	1977087	PRJNA946291
SAMN37499517	Yojoa_06_2021_R_epi_A_bin.54	Yojoa_06_2021_R_epi_A_bin.54	Bacteroidota bacterium	1898104	PRJNA946291
SAMN37499518	Yojoa_06_2021_R_epi_A_bin.62	Yojoa_06_2021_R_epi_A_bin.62	Actinomycetota bacterium	2900548	PRJNA946291
SAMN37499519	Yojoa_06_2021_R_epi_A_bin.65	Yojoa_06_2021_R_epi_A_bin.65	Pseudomonadota bacterium	1977087	PRJNA946291
SAMN37499520	Yojoa_06_2021_R_epi_A_bin.67	Yojoa_06_2021_R_epi_A_bin.67	Planctomycetota bacterium	2026780	PRJNA946291
SAMN37499521	Yojoa_06_2021_R_epi_A_bin.70	Yojoa_06_2021_R_epi_A_bin.70	Planctomycetota bacterium	2026780	PRJNA946291
SAMN37499522	Yojoa_06_2021_R_epi_A_bin.71	Yojoa_06_2021_R_epi_A_bin.71	Pseudomonadota bacterium	1977087	PRJNA946291
SAMN37499523	Yojoa_06_2021_R_epi_A_bin.73	Yojoa_06_2021_R_epi_A_bin.73	Verrucomicrobiota bacterium	2026799	PRJNA946291
SAMN37499524	Yojoa_06_2021_R_epi_A_bin.84	Yojoa_06_2021_R_epi_A_bin.84	Verrucomicrobiota bacterium	2026799	PRJNA946291
SAMN37499525	Yojoa_06_2021_R_epi_A_bin.88	Yojoa_06_2021_R_epi_A_bin.88	Acidobacteriota bacterium	1978231	PRJNA946291
SAMN37499526	Yojoa_06_2021_R_epi_A_bin.99	Yojoa_06_2021_R_epi_A_bin.99	Cyanobacteriota bacterium	2847862	PRJNA946291
SAMN37499527	Yojoa_06_2021_R_epi_K_bin.114	Yojoa_06_2021_R_epi_K_bin.114	Pseudomonadota bacterium	1977087	PRJNA946291
SAMN37499528	Yojoa_06_2021_R_epi_K_bin.28	Yojoa_06_2021_R_epi_K_bin.28	Pseudomonadota bacterium	1977087	PRJNA946291
SAMN37499529	Yojoa_06_2021_R_epi_K_bin.36	Yojoa_06_2021_R_epi_K_bin.36	Actinomycetota bacterium	2900548	PRJNA946291
SAMN37499530	Yojoa_06_2021_R_epi_K_bin.37	Yojoa_06_2021_R_epi_K_bin.37	Planctomycetota bacterium	2026780	PRJNA946291
SAMN37499531	Yojoa_06_2021_R_epi_K_bin.4	Yojoa_06_2021_R_epi_K_bin.4	Bacteroidota bacterium	1898104	PRJNA946291
SAMN37499532	Yojoa_06_2021_R_epi_K_bin.47	Yojoa_06_2021_R_epi_K_bin.47	Actinomycetota bacterium	2900548	PRJNA946291
SAMN37499533	Yojoa_06_2021_R_epi_K_bin.62	Yojoa_06_2021_R_epi_K_bin.62	Actinomycetota bacterium	2900548	PRJNA946291
SAMN37499534	Yojoa_06_2021_R_epi_K_bin.79	Yojoa_06_2021_R_epi_K_bin.79	Pseudomonadota bacterium	1977087	PRJNA946291
SAMN37499535	Yojoa_06_2021_R_epi_K_bin.86	Yojoa_06_2021_R_epi_K_bin.86	Bacteroidota bacterium	1898104	PRJNA946291
SAMN37499536	Yojoa_06_2021_R_hypo_A_bin.110	Yojoa_06_2021_R_hypo_A_bin.110	Planctomycetota bacterium	2026780	PRJNA946291
SAMN37499537	Yojoa_06_2021_R_hypo_A_bin.117	Yojoa_06_2021_R_hypo_A_bin.117	Chloroflexota bacterium	2026724	PRJNA946291
SAMN37499538	Yojoa_06_2021_R_hypo_A_bin.13	Yojoa_06_2021_R_hypo_A_bin.13	Pseudomonadota bacterium	1977087	PRJNA946291
SAMN37499539	Yojoa_06_2021_R_hypo_A_bin.131	Yojoa_06_2021_R_hypo_A_bin.131	Verrucomicrobiota bacterium	2026799	PRJNA946291
SAMN37499540	Yojoa_06_2021_R_hypo_A_bin.134	Yojoa_06_2021_R_hypo_A_bin.134	Verrucomicrobiota bacterium	2026799	PRJNA946291
SAMN37499541	Yojoa_06_2021_R_hypo_A_bin.139	Yojoa_06_2021_R_hypo_A_bin.139	Verrucomicrobiota bacterium	2026799	PRJNA946291
SAMN37499542	Yojoa_06_2021_R_hypo_A_bin.145	Yojoa_06_2021_R_hypo_A_bin.145	Bacillota bacterium	1879010	PRJNA946291
SAMN37499543	Yojoa_06_2021_R_hypo_A_bin.151	Yojoa_06_2021_R_hypo_A_bin.151	Candidatus Parcubacteria bacterium	2762014	PRJNA946291

SAMN37499544	Yojoa_06_2021_R_hypo_A_bin.154	Yojoa_06_2021_R_hypo_A_bin.154	Verrucomicrobiota bacterium	2026799	PRJNA946291
SAMN37499545	Yojoa_06_2021_R_hypo_A_bin.157	Yojoa_06_2021_R_hypo_A_bin.157	Verrucomicrobiota bacterium	2026799	PRJNA946291
SAMN37499546	Yojoa_06_2021_R_hypo_A_bin.161	Yojoa_06_2021_R_hypo_A_bin.161	Candidatus Parcubacteria bacterium	2762014	PRJNA946291
SAMN37499547	Yojoa_06_2021_R_hypo_A_bin.165	Yojoa_06_2021_R_hypo_A_bin.165	Pseudomonadota bacterium	1977087	PRJNA946291
SAMN37499548	Yojoa_06_2021_R_hypo_A_bin.168	Yojoa_06_2021_R_hypo_A_bin.168	Planctomycetota bacterium	2026780	PRJNA946291
SAMN37499549	Yojoa_06_2021_R_hypo_A_bin.27	Yojoa_06_2021_R_hypo_A_bin.27	Planctomycetota bacterium	2026780	PRJNA946291
SAMN37499550	Yojoa_06_2021_R_hypo_A_bin.3	Yojoa_06_2021_R_hypo_A_bin.3	Pseudomonadota bacterium	1977087	PRJNA946291
SAMN37499551	Yojoa_06_2021_R_hypo_A_bin.36	Yojoa_06_2021_R_hypo_A_bin.36	Pseudomonadota bacterium	1977087	PRJNA946291
SAMN37499552	Yojoa_06_2021_R_hypo_A_bin.37	Yojoa_06_2021_R_hypo_A_bin.37	Candidatus Parcubacteria bacterium	2762014	PRJNA946291
SAMN37499553	Yojoa_06_2021_R_hypo_A_bin.39	Yojoa_06_2021_R_hypo_A_bin.39	Verrucomicrobiota bacterium	2026799	PRJNA946291
SAMN37499554	Yojoa_06_2021_R_hypo_A_bin.44	Yojoa_06_2021_R_hypo_A_bin.44	Planctomycetota bacterium	2026780	PRJNA946291
SAMN37499555	Yojoa_06_2021_R_hypo_A_bin.57	Yojoa_06_2021_R_hypo_A_bin.57	Candidatus Parcubacteria bacterium	2762014	PRJNA946291
SAMN37499556	Yojoa_06_2021_R_hypo_A_bin.62	Yojoa_06_2021_R_hypo_A_bin.62	Pseudomonadota bacterium	1977087	PRJNA946291
SAMN37499557	Yojoa_06_2021_R_hypo_A_bin.63	Yojoa_06_2021_R_hypo_A_bin.63	Planctomycetota bacterium	2026780	PRJNA946291
SAMN37499558	Yojoa_06_2021_R_hypo_A_bin.77	Yojoa_06_2021_R_hypo_A_bin.77	Verrucomicrobiota bacterium	2026799	PRJNA946291
SAMN37499559	Yojoa_06_2021_R_hypo_K_bin.10	Yojoa_06_2021_R_hypo_K_bin.10	Methanomicrobiales archaeon	2184052	PRJNA946291
SAMN37499560	Yojoa_06_2021_R_hypo_K_bin.103	Yojoa_06_2021_R_hypo_K_bin.103	Chloroflexota bacterium	2026724	PRJNA946291
SAMN37499561	Yojoa_06_2021_R_hypo_K_bin.110	Yojoa_06_2021_R_hypo_K_bin.110	Bacteroidota bacterium	1898104	PRJNA946291
SAMN37499562	Yojoa_06_2021_R_hypo_K_bin.112	Yojoa_06_2021_R_hypo_K_bin.112	Pseudomonadota bacterium	1977087	PRJNA946291
SAMN37499563	Yojoa_06_2021_R_hypo_K_bin.120	Yojoa_06_2021_R_hypo_K_bin.120	Chloroflexota bacterium	2026724	PRJNA946291
SAMN37499564	Yojoa_06_2021_R_hypo_K_bin.123	Yojoa_06_2021_R_hypo_K_bin.123	Chloroflexota bacterium	2026724	PRJNA946291
SAMN37499565	Yojoa_06_2021_R_hypo_K_bin.139	Yojoa_06_2021_R_hypo_K_bin.139	Actinomycetota bacterium	2900548	PRJNA946291
SAMN37499566	Yojoa_06_2021_R_hypo_K_bin.140	Yojoa_06_2021_R_hypo_K_bin.140	Chloroflexota bacterium	2026724	PRJNA946291
SAMN37499567	Yojoa_06_2021_R_hypo_K_bin.161	Yojoa_06_2021_R_hypo_K_bin.161	Verrucomicrobiota bacterium	2026799	PRJNA946291
SAMN37499568	Yojoa_06_2021_R_hypo_K_bin.162	Yojoa_06_2021_R_hypo_K_bin.162	Verrucomicrobiota bacterium	2026799	PRJNA946291
SAMN37499569	Yojoa_06_2021_R_hypo_K_bin.164	Yojoa_06_2021_R_hypo_K_bin.164	Pseudomonadota bacterium	1977087	PRJNA946291
SAMN37499570	Yojoa_06_2021_R_hypo_K_bin.165	Yojoa_06_2021_R_hypo_K_bin.165	Fusobacteriota bacterium	2060921	PRJNA946291
SAMN37499571	Yojoa_06_2021_R_hypo_K_bin.176	Yojoa_06_2021_R_hypo_K_bin.176	Planctomycetota bacterium	2026780	PRJNA946291
SAMN37499572	Yojoa_06_2021_R_hypo_K_bin.39	Yojoa_06_2021_R_hypo_K_bin.39	Pseudomonadota bacterium	1977087	PRJNA946291
SAMN37499573	Yojoa_06_2021_R_hypo_K_bin.53	Yojoa_06_2021_R_hypo_K_bin.53	Cyanobacteriota bacterium	2847862	PRJNA946291
SAMN37499574	Yojoa_06_2021_R_hypo_K_bin.76	Yojoa_06_2021_R_hypo_K_bin.76	Verrucomicrobiota bacterium	2026799	PRJNA946291
SAMN37499575	Yojoa_06_2021_R_hypo_K_bin.83	Yojoa_06_2021_R_hypo_K_bin.83	Planctomycetota bacterium	2026780	PRJNA946291
SAMN37499576	Yojoa_06_2021_R_hypo_K_bin.87	Yojoa_06_2021_R_hypo_K_bin.87	Verrucomicrobiota bacterium	2026799	PRJNA946291
SAMN37499577	Yojoa_06_2021_R_hypo_K_bin.89	Yojoa_06_2021_R_hypo_K_bin.89	Verrucomicrobiota bacterium	2026799	PRJNA946291
SAMN37499578	Yojoa_06_2021_R_hypo_K_bin.94	Yojoa_06_2021_R_hypo_K_bin.94	Bacteroidota bacterium	1898104	PRJNA946291
